# Nanoparticles for Directed Immunomodulation: Mannose-Functionalized Glycodendrimers Induce Interleukin-8 in Myeloid Cell Lines

**DOI:** 10.1021/acs.biomac.1c00476

**Published:** 2021-07-21

**Authors:** Izabela Jatczak-Pawlik, Michał Gorzkiewicz, Maciej Studzian, Robin Zinke, Dietmar Appelhans, Barbara Klajnert-Maculewicz, Łukasz Pułaski

**Affiliations:** † Department of Hypertension, Chair of Nephrology and Hypertension, Medical University of Lodz, 281/289 Rzgowska Street, Lodz 93-338, Poland; ‡ Polish Mother’s Memorial Hospital Research Institute (PMMHRI), 281/289 Rzgowska Street, Lodz 93-338, Poland; § Department of General Biophysics, Faculty of Biology and Environmental Protection, University of Lodz, 141/143 Pomorska Street, Lodz 90-236, Poland; ∥ Department of Molecular Biophysics, Faculty of Biology and Environmental Protection, University of Lodz, 141/143 Pomorska Street, Lodz 90-236, Poland; ⊥ Leibniz Institute of Polymer Research Dresden, Hohe Straße 6, Dresden 01069, Germany; # Laboratory of Transcriptional Regulation, Institute of Medical Biology PAS, 106 Lodowa Street, Lodz 93-232, Poland

## Abstract

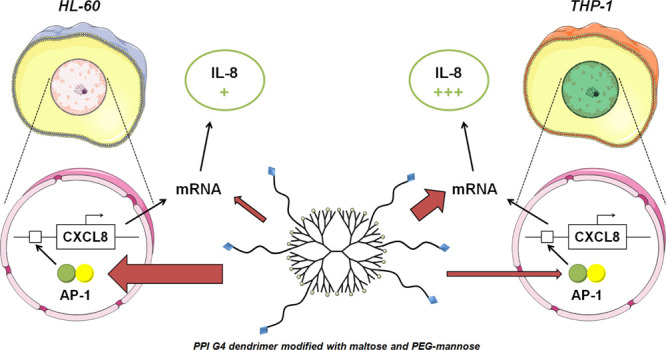

New therapeutic strategies for personalized medicine need to involve innovative pharmaceutical tools, for example, modular nanoparticles designed for direct immunomodulatory properties. We synthesized mannose-functionalized poly(propyleneimine) glycodendrimers with a novel architecture, where freely accessible mannose moieties are presented on poly(ethylene glycol)-based linkers embedded within an open-shell maltose coating. This design enhanced glycodendrimer bioactivity and led to complex functional effects in myeloid cells, with specific induction of interleukin-8 expression by mannose glycodendrimers detected in HL-60 and THP-1 cells. We concentrated on explaining the molecular mechanism of this phenomenon, which turned out to be different in both investigated cell lines: in HL-60 cells, transcriptional activation via AP-1 binding to the promoter predominated, while in THP-1 cells (which initially expressed less IL-8), induction was mediated mainly by mRNA stabilization. The success of directed immunomodulation, with synthetic design guided by assumptions about mannose-modified dendrimers as exogenous regulators of pro-inflammatory chemokine levels, opens new possibilities for designing bioactive nanoparticles.

## Introduction

Virtually all clinical treatments affect the immunological system in ways that can strongly vary from patient to patient, driving the development of directed immunomodulators for a personalized medicine setting. Novel biofunctional nanoparticles are a promising tool for directed immunomodulation, where specific intracellular mechanisms in immune cells are activated or repressed to obtain potentially clinically significant outcomes (immunostimulation or immunosuppression). In recent years, sugar-modified dendrimers (glycodendrimers) became one of the most promising classes of highly branched dendritic polymers with several potential biomedical applications.^1^ At this point, it is crucial to note that specific carbohydrate–protein interactions are involved in numerous cellular processes, including bacterial and viral adhesion, regulation of cell growth and differentiation, and most importantly—immunomodulation.^2,3^ Therefore, the insightful characterization and understanding of molecular mechanisms by which glycodendrimers impact the cellular homeostasis may enable new clinical applications for this type of nanoparticles, for example, as modulators triggering the anticancer activity of the innate immune system. The great variety of both dendritic scaffolds and carbohydrates gives the opportunity to develop different branched glycopolymers with unique properties. This creates several possibilities for specific immunomodulation in pharmacology, enabling controlled inhibition or activation of immune cell proliferation, cytokine release, or antibody secretion without detrimental side effects associated with the currently available chemical immunomodulators.^4^

We have embarked on a multipronged study of poly(propyleneimine) (PPI) glycodendrimers as potential immunomodulatory substances, concentrating on the molecular mechanisms of immunomodulation in cellular models (in vitro cultured cell lines). We were previously able to validate the idea of affecting important signaling pathways in immune cells by sugar-functionalized dendrimers^5^ and to demonstrate the importance of different sugar moieties for these biological effects.^6^ As a logical next step, we extended the study to mannose, a monosaccharide that is exceptionally important to the immune system. While immunomodulatory applications of mannose-modified dendrimers have been previously published (e.g., mannose as an adjuvant for dendrimeric vaccines^7^ or as a disruptor of bacterial biofilm formation^8^), our focus remains on mechanistic studies of cellular processes (signaling and gene regulation) impacted by glycodendrimer treatment.

Since the known effects of mannose-containing macromolecules, especially in the immune system, are mediated mostly by glycan–protein (receptor) interactions, the steric environment of the mannose moiety is especially important in studies involving artificial nanoparticles. Therefore, while building upon the previous body of work performed on PPI glycodendrimers with simple and complex sugars attached directly to the distal amino groups, in the present study, we decided to design a novel chemical mode of modular attachment of mannose moieties, with the novelty consisting of designing a PEG-based linker of a length ensuring optimal flexibility and steric accessibility as well as of the application of click chemistry for adding mannose residues in an orthogonal manner to previous glyco-modifications. In this manner, it was possible to make use of formerly characterized open-shell maltose-decorated dendrimers, which were the basis of our previous studies on immunomodulation.

Our research focused on interleukin-8 (IL-8), the mRNA expression of which has been shown to increase significantly in cells stimulated with glycodendrimers.^6^ IL-8 is an interesting immune mediator, which is best known as one of the most important pro-inflammatory chemokines, produced at infection sites to attract and activate granulocytes.^9,10^ However, it has a much more complex role as a regulatory cytokine within the differentiating myeloid lineage, where it is produced in response to pro-inflammatory stimuli and affects differentially various terminally differentiated cell types, for example, stimulating oxidative burst and extrusion of extracellular traps in neutrophils,^11^ phagocytosis in macrophages,^12^ as well as immunosuppressive function in myeloid-derived suppressor cells.^13^ Its production is under tight control, with constitutive expression restricted to less differentiated cells^14^ and in other cell types stimulated upon crossing a certain activation threshold, for example, by Toll-like receptor ligand binding in mature macrophages.^15^ Taking all these aspects into consideration, it can be concluded that the mechanisms of IL-8 biogenesis are at the nexus of early myeloid cell function and commitment to differentiation. Therefore, immunomodulation by influencing IL-8 expression (as demonstrated for mannose glycodendrimers in the present study) is important not just for the process of effector cell chemotaxis in inflammation but also in preparing a cellular background for immune response with regard to the myeloid cell repertoire.

## Experimental Section

### Dendrimers

**Figure 1:**
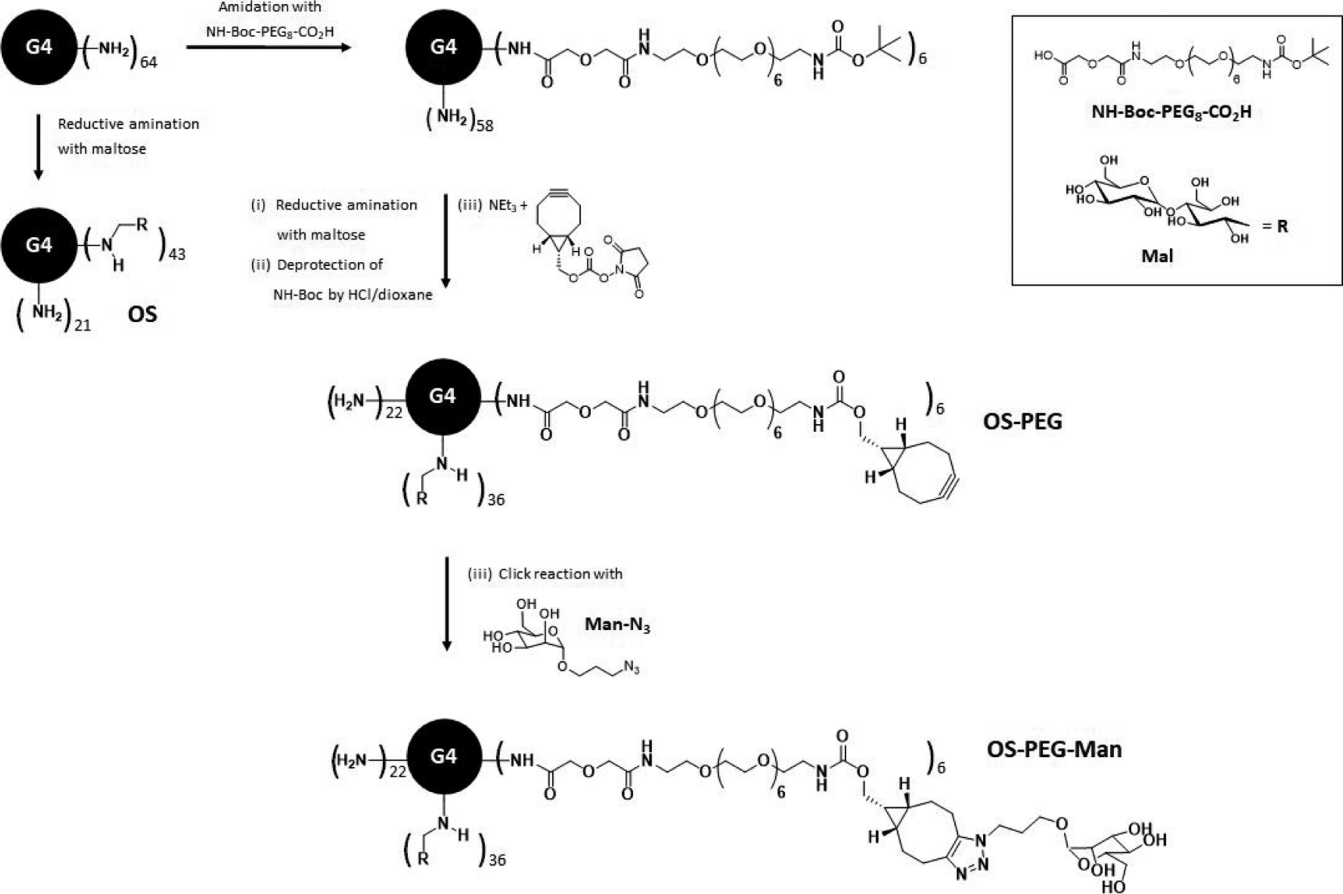
Simplified scheme presenting the subsequent steps of the synthesis of investigated nanoparticles.

PPI dendrimer of the 4th generation* with 64 primary amino surface groups was obtained from Symo-Chem (Eindhoven, Netherlands) and served as the starting point for further synthesis of three glycodendrimer species for further use in biological assays: OS (PPI with 43 maltose moieties on the surface), OS-PEG (an analogous molecule with 6 PEG-based linkers prepared for mannose attachment), and OS-PEG-Man (OS-PEG with mannose moieties attached to PEG linkers). Figure 1 presents the chemical structures of the respective molecules. Detailed chemical synthesis procedure and characterization are presented in the Supporting Information.

*According to Tomalia and Rookmaker,^16^ the nomenclature for Tomalia-type PAMAM dendrimers can be used for PPI dendrimers. Hence, we adopted the suggested classification, describing commercially available PPI dendrimer of the 5th generation (DAB-Am-64) as 4th generation.

### Size and Zeta Potential Measurements

Measurements of size and zeta potential were performed with the use of a Zetasizer Nano ZS (Malvern Instruments Ltd., Malvern, UK). Water and phosphate-buffered saline (PBS) solutions containing the studied compounds at the final dendrimer concentration of 10 μM were placed in low-volume sizing cuvettes (ZEN0112, Malvern Instruments Ltd., Malvern, UK) for size determination or in folded capillary cells (DTS 1070, Malvern Instruments Ltd., Malvern, UK) for zeta potential measurements and measured at 25 °C. The data were analyzed using the Malvern software.

### Cell Culture

THP-1 (acute monocytic leukemia) and HL-60 (acute promyelocytic leukemia) human cell lines were purchased from ATCC (Manassas, VA, USA) and maintained under standard conditions in RPMI-1640 Medium (Thermo Fisher Scientific, Waltham, MA, USA) supplemented with 10% fetal bovine serum (Sigma-Aldrich, St. Louis, MO, USA) at 37 °C in an atmosphere of 5% CO_2_. The cells were subcultured 3 times per week.

### Cytotoxicity Assay

To estimate the potential cytotoxic activity of glycodendrimers, a neutral red assay was performed.^17^ Cells were seeded into 96-well transparent plates precoated with poly-l-lysine at a density of 1 × 10^4^ cells per well and treated with increasing concentrations of dendrimers (0.16–5 μM) for 2.5 and 6 h. Following incubation, the cells were centrifuged, washed with PBS, and incubated with 50 μg/mL neutral red in Hanks' balanced salt solution for 3 h at 37 °C. Subsequently, the cells were washed again with PBS, lyzed in 50% ethanol, 1% acetic acid, and the absorbance of neutral red internalized by lysosomes of viable cells was measured at 550 nm using an EnVision plate reader (PerkinElmer, Waltham, MA, USA). Cell viability was calculated as the percentage of neutral red uptake by cells in the untreated control.

### Gene Expression Assay

The gene expression level was determined by quantitative real-time reverse transcription-polymerase chain reaction (RT-PCR). Aliquots of 1 × 10^6^ of HL-60 and THP-1 cells were cultured for up to 6 h with glycodendrimers at final concentrations of 5 μM. For mRNA stability experiments, cells were pretreated for 2 h with 5 μg/mL actinomycin D to stop transcription and subsequently cultured with glycodendrimers. Following incubation, cells were harvested and washed once with PBS. For selected experiments, the cells were pretreated with signaling pathway inhibitors (10 μM, 30 min). Total cellular RNA was isolated using TRI Reagent (Sigma-Aldrich, St. Louis, MO, USA) according to the manufacturer’s protocol. Complementary DNA (cDNA) was transcribed from mRNA using a High Capacity cDNA Reverse Transcription Kit (Thermo Fisher Scientific, Waltham, MA, USA) and used for real-time PCR amplification with the GoTaq qPCR Master Mix (Promega, Madison, WI, USA) according to manufacturer’s protocol, with 0.25 μM concentration of forward and reverse intron-spanning primers (for primer sequences, see Table 1). The reference genes (*HPRT1*, *HMBS*, and *TBP*) were selected according to the GeNorm procedure.^18^ PCR reactions were performed in 96-well microplates using the CFX96 Real-Time PCR Detection System (Bio-Rad Laboratories, Inc., Hercules, CA, USA). The expression level of the assayed genes was calculated by the ΔΔ*Ct* method and expressed as the number of cognate mRNA copies per 1000 copies of geometric-averaged mRNA for reference genes.

**Table 1:** Primer Sequences

gene	forward and reverse sequences (5′–3′)
*HPRT1*	Fw: TGACACTGGCAAAACAATGCA
	Rv: GGTCCTTTTCACCAGCAAGCT
*HMBS*	Fw: GGCAATGCGGCTGCAA
	Rv: GGGTACCCACGCGAATCAC
*TBP*	Fw: CACGAACCACGGCACTGATT
	Rv: TTTTCTTGCTGCCAGTCTGGAC
*CXCL8*	Fw: CCTTGGCAAAACTGCACCTT
	Rv: CTGGCCGTGGCTCTCTTG
*CD14*	Fw: GCCGCTGTGTAGGAAAGAAG
	Rv: AGGTTCGGAGAAGTTGCAGA
*CD69*	Fw: GCAACCTTTGGATGCACTTT
	Rv: ATGCATGAAGGGCTCTCACT
*F3*	Fw: TTGGCAAGGACTTAATTTATAC
	Rv: CTGTTCGGGAGGGAATCAC
*FOS*	Fw: CTGGCGTTGTGAAGACCAT
	Rv: TCCCTTCGGATTCTCCTTTT
*MYC*	Fw: CCTGGTGCTCCATGAGGAGAC
	Rv: CAGACTCTGACCTTTTGCCAGG
*PER2*	Fw: AGCTGCTTGGACAGCGTCATCA
	Rv: CCTTCCGCTTATCACTGGACCT
*NR1D1*	Fw: CTGCCAGCAATGTCGCTTCAAG
	Rv: TGGCTGCTCAACTGGTTGTTGG

### Cytokine Assay

Cultured HL-60 and THP-1 cells were stimulated for 6 h with tested dendrimers at 5 μM concentration. Subsequently, cells were removed by centrifugation (5 min, 5000×g, RT) and protein concentration of IL-8 was measured in the supernatants using Quantikine ELISA kits (R&D Systems, Inc., Minneapolis, MN, USA). The assay was performed strictly according to the manufacturer’s protocol, the absorbance was read in an EnVision plate reader (PerkinElmer, Waltham, MA, USA) at 450 nm. Data were presented as the absolute concentration in conditioned medium, calculated from a calibration curve included with the assay kit.

### In-Cell Western Blot

THP-1 and HL-60 cells were stimulated for 2.5 h with glycodendrimers at a final concentration of 5 μM. Aliquots of 1 × 10^5^ cells were then withdrawn from the culture and transferred to a thin-bottom 96-well plate coated previously with poly-l-lysine. After 10 min of sedimentation at 37 °C, the cells were centrifuged (5 min, 100×*g*, RT) to enhance cell adhesion to the plate. Following gentle aspiration of the culture medium and a single wash with PBS, the cells were immediately fixed for 20 min at RT with PBS-buffered 2% formaldehyde solution (pH 7.2) freshly prepared from paraformaldehyde and incubated with blocking buffer [PBS, 3% bovine serum albumin (BSA)] for 30 min at RT. Subsequently, the cells were incubated with primary antibodies overnight at RT: anti-c-Jun antibody [E254] (ab32137) or anti-JunD (phospho S100) + c-Jun (phospho S73) antibody [EPR16586] (ab178858) (Abcam, Cambridge, UK), 1:300 in PBS with 1% BSA and 0.1% Tween 20. Following incubation, the cells were washed 3 times with PBS with 0.1% Tween 20 and incubated with a secondary antibody: Goat anti-Rabbit IgG H&L (IRDye 800CW) pre-adsorbed (ab216773) (Abcam, Cambridge, UK), 1:500 in PBS containing 1% BSA, 0.1% Tween 20, and 0.2 μM CellTag 700 Stain for 1 h at RT. Subsequently, the cells were washed 3 times with PBS with 0.1% Tween 20, and 100 μL of PBS was added to each well. The antibody–protein complexes were visualized on an Odyssey IR imager (LI-COR Biosciences, Lincoln, NE, USA). Ratios of bound anti-phospho-c-Jun antibody to anti-total-c-Jun antibody were averaged and normalized for the number of cells per well. Data were presented as a percentage of the control (untreated) phosphorylation ratio.

### DNA-Binding ELISA for AP-1

Cultured HL-60 and THP-1 cells were stimulated for 2.5 h with tested dendrimers at 5 μM concentration. Subsequently, the cells were collected, and then the nuclear extracts were prepared and assay performed with the use of a TransAM AP-1 Kit (Active Motif Inc., Carlsbad, CA, USA) according to the manufacturer’s protocol. The absorbance was read in an EnVision plate reader (PerkinElmer, Waltham, MA, USA) at 450 nm with a reference wavelength of 655 nm. Data were expressed as antibody-linked HRP enzymatic activity in units of absorbance.

### Electrophoretic Mobility Shift Assay

Aliquots of 1 × 10^6^ THP-1 and HL-60 cells were cultured for 2.5 h with glycodendrimers at a final concentration of 5 μM. Following incubation, the cells were washed once with PBS and centrifuged at 500×*g* for 3 min. The supernatant was carefully removed, leaving the cell pellet as dry as possible. Nuclear extracts were then prepared using the NE-PER Nuclear and Cytoplasmic Extraction Reagents (Thermo Fisher Scientific, Waltham, MA, USA) with the Halt Protease and Phosphatase Inhibitor Cocktail (Thermo Fisher Scientific, Waltham, MA, USA) according to the manufacturer’s recommendation. Protein concentration of the extracts was determined using a Microplate BCA Protein Assay Kit-Reducing Agent Compatible (Thermo Fisher Scientific, Waltham, MA, USA), and aliquots were frozen at −80 °C until use.

Nuclear extracts were analyzed for the presence of active (DNA-binding) AP-1 using double-stranded oligonucleotide probes with the consensus binding sequences, labeled with IRDye 700 infrared fluorescence dye (5′-GTG TGA TGA CTC AGG TTT G-3′, consensus sites are underlined), custom-synthesized by Metabion International AG (Planegg, Germany). Extracts were incubated for 30 min at 4 °C with 0.5 μg/mL salmon sperm DNA in a binding buffer: 5% glycerol, 10 mM MgCl_2_, 1 mM DTT, 50 mM NaCl, 0.1% NP-40, 0.4 μM ZnCl_2_, and 10 mM Tris-HCI, pH 8 with or without the addition of 2 pmol/μL of the competing, unstained oligonucleotide probe. After this time, labeled AP-1 probe was added to the mixture at the final concentration of 0.02 pmol/μL and further incubated for 30 min at 4 °C. DNA-protein complexes were analyzed by electrophoresis in non-denaturing conditions on a 12% polyacrylamide gel at 4 °C. The probe-protein complexes were visualized on an Odyssey IR imager (LI-COR Biosciences, Lincoln, NE, USA). Band intensities were quantified digitally using ImageJ software.

### Statistics

For statistical significance testing for single pairwise comparisons, Student’s *t*-test was applied. For multiple comparisons, ANOVA followed by Tukey’s post-hoc test was applied. In all tests, *p* values <0.05 were considered to be statistically significant. Data are presented as arithmetic mean ± standard deviation (SD).

## Results

### Biophysical Characteristics of PPI Glycodendrimers

For the application of nanoparticles in cellular systems, it is important to characterize molecular-level properties which impact the interactions between individual particles and the cell surface. Thus, we determined the hydrodynamic diameter (indicating particle size) and zeta potential (indicating surface charge distribution and dispersion propensity in solution) for the three assayed nanoparticle types (Table 2). The obtained values showed that all tested glycodendrimers have sizes in the range predicted for globular conformation (as is typical for most dendrimers of higher generations). A strongly positive zeta potential in aqueous solution is indicative of relatively good accessibility of remaining free amino groups in the open-shell architecture, while its strong decrease with buffering to a near-neutral pH suggests the lack of propensity to form very strong, disruptive interactions with negatively charged cell membrane components, providing a rationale for decreased acute toxicity in comparison to unmodified PPI dendrimers. The fact that all three glycodendrimer species show similar values of zeta potential at physiological pH allowed us to use the intermediate synthesis steps (OS and OS-PEG macromolecules) as biological activity controls for the main assayed macromolecule, OS-PEG-Man, since in this case, most differences in dendrimer–cell interactions for these compounds are expected to be based on affinity toward specific membrane receptors, rather than on crude nonspecific biophysical effects.

**Table 2:** Results of Size and Zeta Potential Measurements for Investigated Nanoparticles^a^

		OS	OS-PEG	OS-PEG-Man
hydrodynamic diameter [nm]		6.27 ± 0.26	16.89 ± 1.43	18.78 ± 3.07
zeta potential [mV]	H_2_O	34.09 ± 2.94	35.5 ± 2.51	33.02 ± 0.74
	PBS	4.67 ± 0.81	4.5 ± 0.13	4.21 ± 0.68

a Hydrodynamic diameter and zeta potential were measured using Zetasizer Nano ZS equipment in 10 μM solutions. Data presented as mean ± SD (*n* = 4)

### Determination of Biocompatible Concentration Range for PPI Glycodendrimers

**Figure 2:**
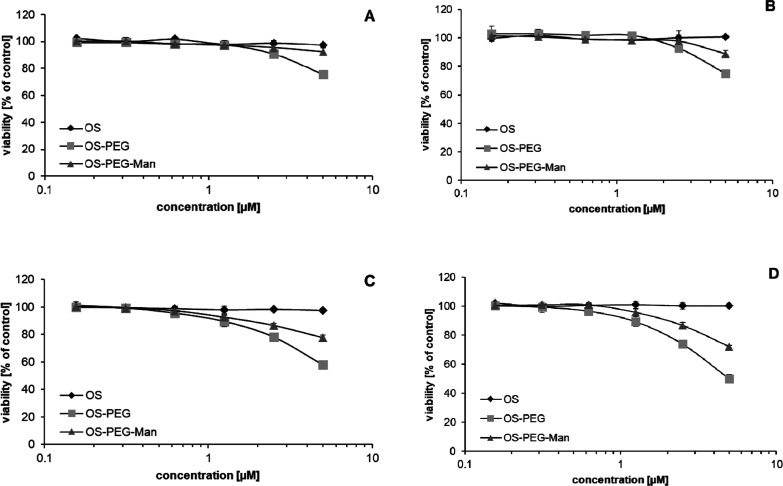
Toxicity toward in vitro cultured cell lines after treatment with investigated nanoparticles. Cells from HL-60 (panels A,C) and THP-1 (panels B,D) cell lines were treated for 2.5 h (panels A,B) and 6 h (panels C,D) with nanoparticles at indicated concentrations, and their viability was tested with the neutral red uptake assay. Data presented as percentage of control (untreated) neutral red uptake rate. Data points correspond to mean ± SD (*n* = 4).

Since previous studies have demonstrated the relatively low toxicity of maltose open-shell glycodendrimers at low micromolar concentrations toward immune system cells,^19,20^ we set out to determine the maximal subtoxic concentration for OS-PEG-Man for subsequent use in immunomodulatory bioactivity tests. Somewhat surprisingly, modification with the PEG linker increased the cytotoxic potential of maltose-modified nanoparticles at the highest tested concentrations, while subsequent mannose attachment to the linker decreased the toxic effect by half (Figure 2). Still, the mannose-modified glycodendrimer was biocompatible at all tested concentrations (with viability exceeding 70% even after 6 h of incubation at the highest concentration). Monocytic lineage THP-1 cells were slightly more vulnerable to this toxic effect than the general myeloid lineage representatives from the HL-60 cell line.

### Induction of IL-8 Expression by OS-PEG-Man

**Figure 3:**
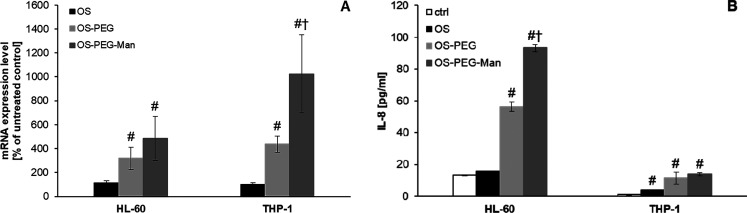
IL-8 expression after treatment with the investigated nanoparticles. Cells from HL-60 and THP-1 cell lines were treated for 6 h with nanoparticles at 5 μM concentration. Subsequently, IL-8 expression was measured at the level of mRNA (real-time RT-PCR after RNA isolation from treated cells) and secreted protein (ELISA for active IL-8 in conditioned medium). IL-8-coding mRNA levels are calculated as the number of cognate mRNA copies per averaged reference gene and presented as the percentage of values for untreated control (mean ± SD, *n* = 4, panel A). IL-8 protein levels in the medium are calculated from the reference ELISA curve and presented as the absolute concentration in the conditioned medium (mean ± SD, *n* = 4, panel B). # Statistically significant difference compared to control at *p* < 0.05. † Statistically significant difference compared to OS-PEG at *p* < 0.05.

Incubation with mannose-decorated glycodendrimers led to an increase in mRNA level for pro-inflammatory IL-8 in both tested cell types, with the effect being stronger in the THP-1 cell line (Figure 3A); this is linked to the fact that the basal mRNA expression levels in these cells, studied and published by us previously,^6^ differ strongly, with *CXCL8* mRNA levels being more than 4 times higher in HL-60 cells. While linker-free OS macromolecules exerted no biological effect at all in this experiment, the control mannose-free OS-PEG also increased the IL-8 mRNA content; still, in THP-1 cells, this effect was significantly less pronounced than for OS-PEG-Man. We confirmed the biological relevance of mRNA changes by assaying IL-8 production and secretion at the protein level by enzyme-linked immunosorbent assay (ELISA), yielding a similar pattern of stimulation (Figure 3B). While the basal production of IL-8 by HL-60 cells was determined to be quite high (in accordance with the literature) and thus reached very high levels upon induction with OS-PEG-Man (on the order of 0.1 ng/mL in conditioned medium), the stimulation ratio was even higher (14-fold vs 7-fold) in THP-1 cells which produce low levels of IL-8 in the unstimulated condition. Here again, control OS-PEG dendrimers had a weaker effect than OS-PEG-Man.

### Mechanisms of *CXCL8* mRNA Upregulation by OS-PEG-Man

**Figure 4:**
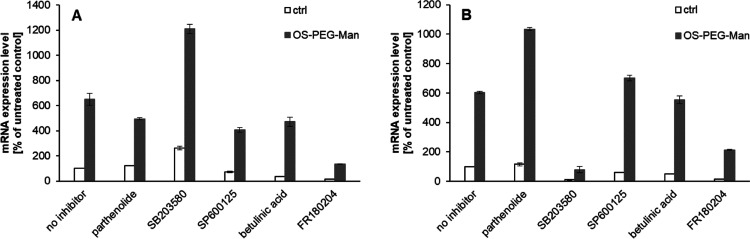
Effect of signaling pathway inhibitors on IL-8 mRNA expression. Cells from HL-60 (panel A) and THP-1 (panel B) cell lines were treated for 6 h with OS-PEG-Man nanoparticles at 5 μM concentration after pretreatment (30 min) with signaling pathway inhibitors (10 μM). IL-8-coding mRNA levels are calculated as the number of cognate mRNA copies per averaged reference gene and presented as a percentage of values for untreated (no nanoparticle, no inhibitor) control (mean ± SD, *n* = 4).

Since the increase in active IL-8 polypeptide secretion was paralleled by its mRNA levels in OS-PEG-Man-stimulated immune lineage cells, we set out to determine the molecular mechanism of mRNA upregulation for the *CXCL8* gene encoding IL-8. Since the *CXCL8* gene is known to be under strong transcriptional control of several ubiquitous transcription factors which respond to environmental signals via cellular signaling pathways, we first verified the involvement of some of these pathways in the observed phenomenon by pharmacological inhibition of pathway progression. In these experiments, cell treatment with the OS-PEG-Man was performed in the presence of specific inhibitors of enzymes or protein–protein interactions required for signal transduction through five distinct pathways known to be involved in *CXCL8* regulation: NF-κB (parthenolide), p38 (SB203580), JNK (SP600125), JAK/STAT3 (betulinic acid), and ERK (FR180204), in order to identify those able to repress the observed effect. The obtained results confirmed the complex role of some of the assayed pathways in *CXCL8* gene regulation in myeloid cell lines but provided no clear answer to questions on OS-PEG-Man action mechanism since those inhibitors, which had a negative or positive effect, had it on both untreated and treated cells (Figure 4). Specifically, the most conspicuous effect was that of p38 inhibition by SB203580 (Figure 4A), causing stimulation of *CXCL8* mRNA expression in HL-60 cells (where it is already high at the beginning) with a further inhibition in THP-1 cells (expressing significantly lower levels of IL-8). On the other hand, ERK inhibition depressed *CXCL8* mRNA in both cell lines. Both of these effects were independent of glycodendrimer stimulation.

**Figure 5:**
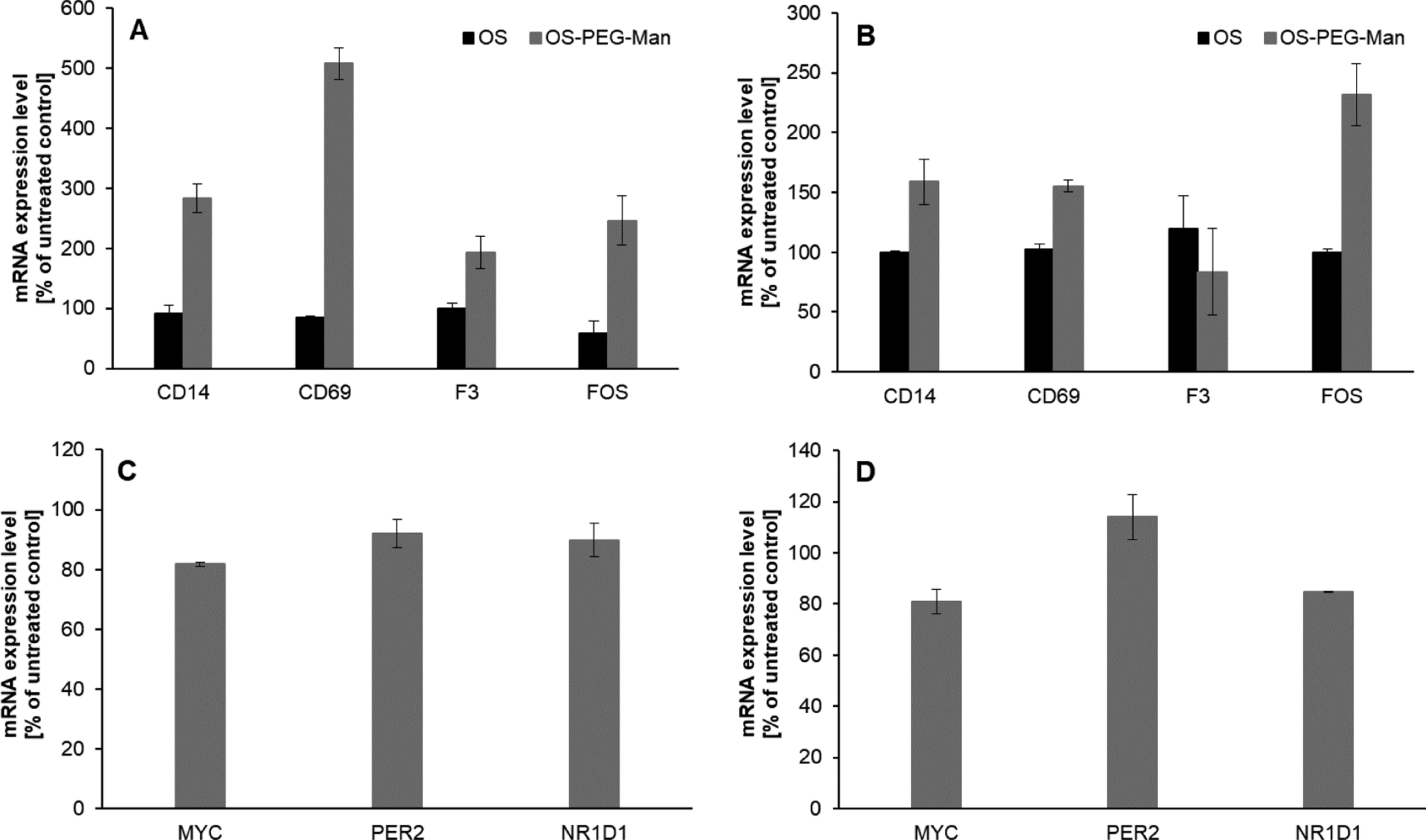
Expression of AP-1 marker genes after treatment with investigated nanoparticles. Cells from HL-60 (panels A,C) and THP-1 (panels B,D) cell lines were treated for 6 h with OS (panels A,B) and OS-PEG-Man (panels A–D) nanoparticles at 5 μM concentration. Subsequently, mRNA levels for investigated genes were measured by real-time RT-PCR after RNA isolation from treated cells. These mRNA levels are calculated as the number of cognate mRNA copies per averaged reference gene and presented as a percentage of values for untreated control (mean ± SD, *n* = 6).

While the involvement of upstream signaling pathway elements in OS-PEG-Man-mediated induction of IL-8 could not be proven, the obvious role of some of these pathways in transcriptional regulation of the basal expression of the *CXCL8* gene led us to verify the involvement of relevant transcription factors in the investigated phenomenon. Since both p38 and ERK pathways often act via the AP-1 transcription factor complex, we initially verified the activity of this complex via the level of mRNA expression of some of its known marker genes in myeloid lineage cells, such as *CD14*, *CD69*, *F3*, and *FOS* (Figure 5A,B). We were able to show that all of these genes are indeed induced by OS-PEG-Man treatment in both cell lines (with the exception of *F3* in THP-1 cells); the mannose-free OS compound had no effect, pointing to a potential role of AP-1 in the OS-PEG-Man effect on IL-8 levels. Conversely, the expression of another transcription factor, c-Myc, another common mediator of p38, and ERK effects in myeloid cells (often downstream of AP-1), as well as that of c-Myc marker genes PER2 and NR1D1, was unaffected by OS-PEG-Man (Figure 5C,D). This indicates a direct role for AP-1 activation in the induction of *CXCL8* transcription rather than acting indirectly through downstream factors.

**Figure 6:**
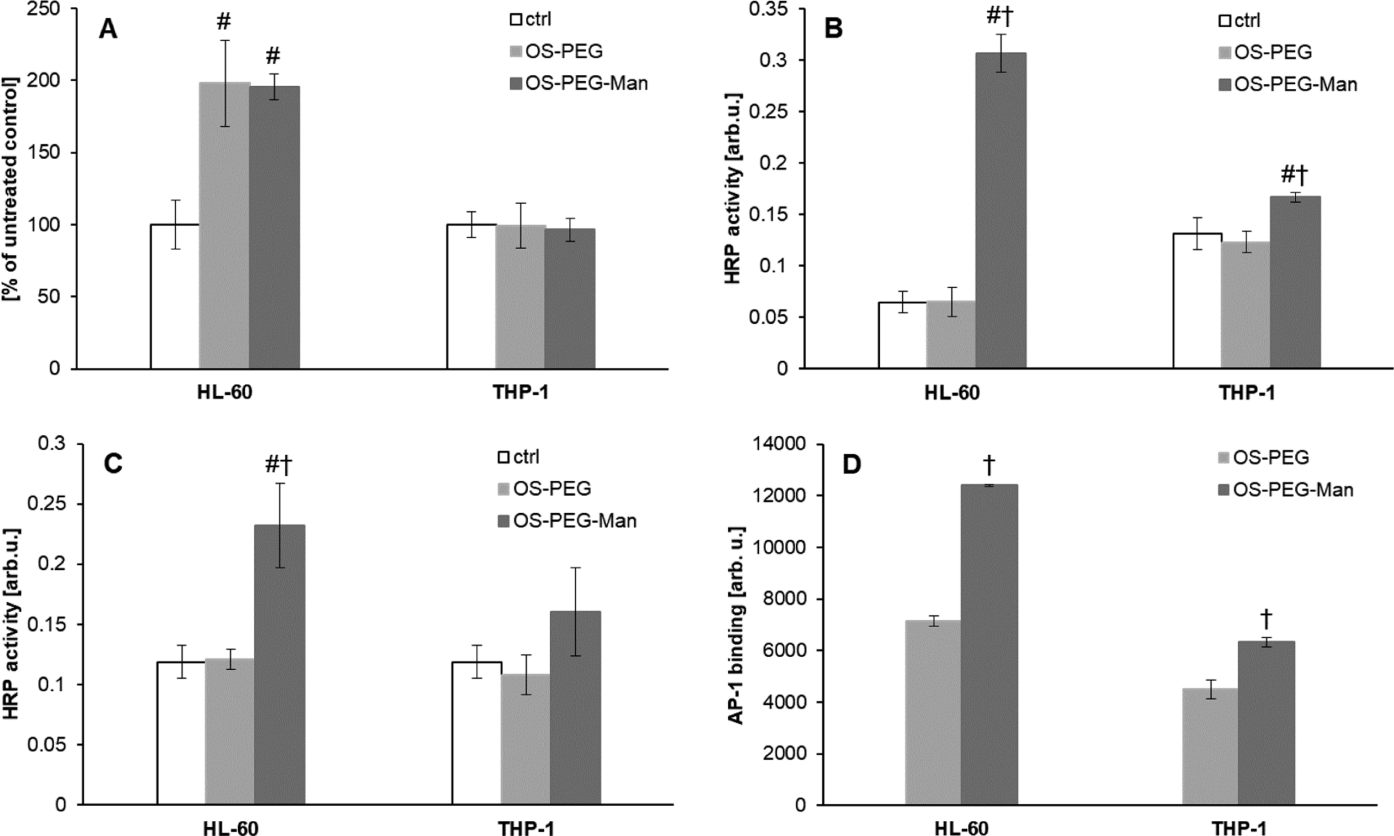
AP-1 activation by treatment with the investigated nanoparticles. Cells from HL-60 and THP-1 cell lines were treated for 2.5 h with nanoparticles at 5 μM concentration. Subsequently, the level of activation of the transcription factor complex AP-1 was assayed by immunofluorescence assay for phosphorylated c-Jun with infrared-fluorescent secondary antibodies (In-Cell Western); ELISA-type DNA binding immunoassay (Trans-AM); and direct DNA binding assay (EMSA). Panel A shows the ratio of the bound anti-phospho-c-Jun antibody to anti-total-c-Jun antibody in whole fixed cells, detected by infrared fluorescence scanning (presented as mean ± SD, *n* = 3). Panels B and C show the amount of specific primary antibodies against phosphorylated c-Jun (B) and c-Fos (C) binding to immobilized DNA oligonucleotide-bound activated AP-1 from cell extracts, detected as enzyme-linked activity (HRP) with a colorimetric substrate (presented as mean ± SD, *n* = 3). Panel D shows the amount of fluorescently labeled oligonucleotide bound specifically to activated AP-1, detected by infrared fluorescence scanning of electrophoretic gel (presented as mean ± SD, *n* = 3). # Statistically significant difference compared to control at *p* < 0.05. † Statistically significant difference compared to OS-PEG at *p* <0.05.

Pursuing this line of evidence, we used various techniques to collect biochemical proof of the stimulation of the AP-1 transactivatory function by OS-PEG-Man. The results were strongly dependent on the cellular background and did not lead to a coherent picture of OS-PEG-Man action: first, we applied a direct immunoassay (In-Cell Western) for phosphorylated c-Jun (AP-1 component) that showed no stimulation of its phosphorylation by glycodendrimers in THP-1 cells, while in HL-60 cells, the effect of OS-PEG-Man and OS-PEG was comparable (Figure 6A). Conversely, an ELISA-type assay for activated AP-1 elements (c-Jun and c-Fos) demonstrated the specific induction of AP-1 DNA-binding activity by OS-PEG-Man, which was much more pronounced in the HL-60 cell line (Figure 6B,C). This was subsequently confirmed by direct electrophoretic mobility shift assay (EMSA) for AP-1 DNA binding, where OS-PEG-Man showed an activating phenotype in HL-60 cells and much less so in THP-1 (Figure 6D).

**Figure 7:**
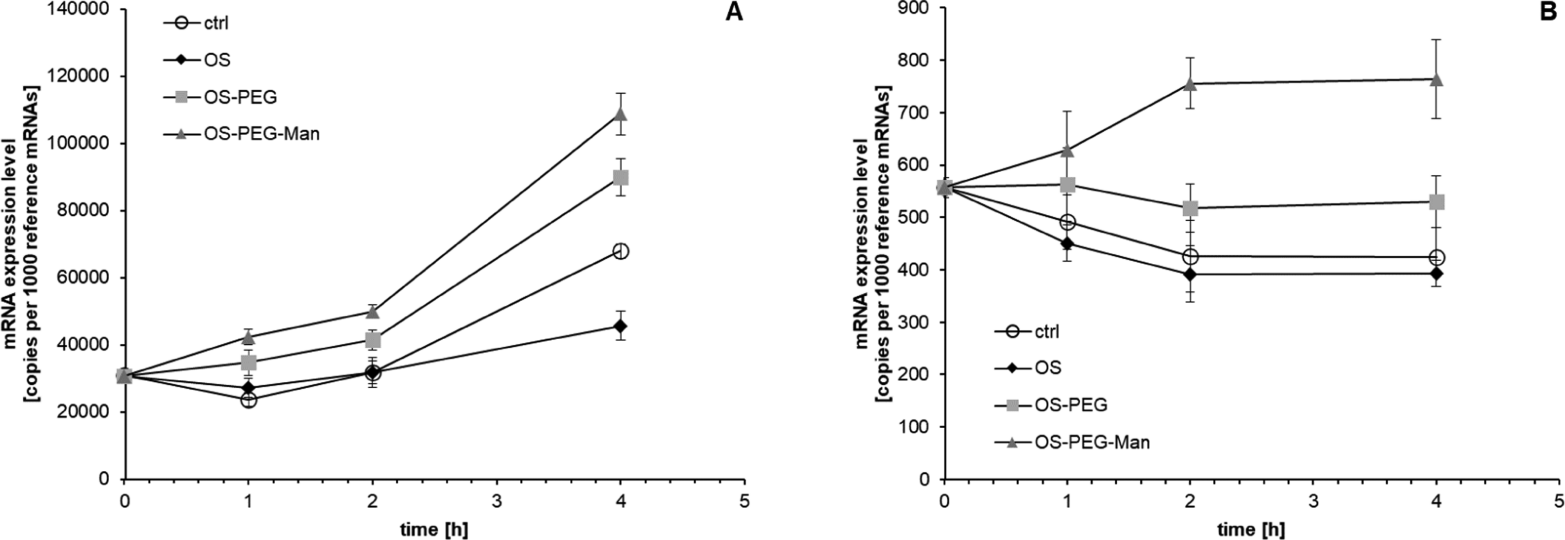
Effect of investigated nanoparticles on IL-8 mRNA stability. Cells from HL-60 (panel A) and THP-1 (panel B) cell lines were treated for 2 h with 5 μg/mL actinomycin D to stop transcription and subsequently incubated for the indicated length of time with nanoparticles at 5 μM concentration (still in the presence of actinomycin D). IL-8-coding mRNA levels are presented as the number of cognate mRNA copies per averaged reference gene (mean ± SD, *n* = 6).

Since specific transcriptional effects were not strong enough to explain the whole extent of IL-8 induction in myeloid cells by OS-PEG-Man, we verified the possible contribution of mRNA stability regulation to this phenomenon. After inhibition of nascent RNA transcription with actinomycin D, the kinetics of IL-8 mRNA decay was found to be differentially regulated by glycodendrimers in both cell lines (Figure 7). In HL-60 cells, IL-8 mRNA was inherently relatively stable (more stable than reference genes), and OS-PEG-Man had some further stabilizing influence but so did, to some extent, OS-PEG, indicating weak specificity for mannose in OS-PEG-Man for this stabilization effect. On the other hand, in THP-1 cells, IL-8 mRNA constitutively degraded at a higher rate than reference genes. Here, OS-PEG-Man had a very strong and pronounced specific stabilizing effect; its extent corresponded to the mRNA induction level seen in Figure 3 and could provide a mechanistic explanation for it.

## Discussion

For almost 40 years, dendrimers have aroused constant interest due to the possibility of their clinical use both as drug carriers and bona fide polymeric therapeutics.^21^ The optimized methods of synthesis and purification allowed us to obtain sphere-shaped polymers with uniform molecular weight and regular, highly branched architecture that provides a number of unique physicochemical and biological properties.^22,23^ The latter is usually affected by the character of highly reactive surface moieties, which in the case of cationic dendrimers [such as most comprehensively studied poly(amidoamine) (PAMAM) or PPI macromolecules] are responsible for nonspecific interactions with negatively charged biological membranes and significant cytotoxicity.^24,25^ Since positive charge may hamper the direct clinical application of dendrimers, intensive studies on covalent modification of their surface are being carried out in order to partially or completely eliminate the cationic nature of the terminal groups.^26^ For this purpose, PEGylation^27,28^ and glycosylation^29,30^ are most commonly performed. The attachment of sugar moieties has been found to lower the cytotoxicity and improve the biocompatibility of cationic dendrimers, additionally prolonging their blood circulation time, modulating biodistribution patterns, and increasing the drug-loading capacity.^29−31^ Carbohydrates may also enhance the cellular recognition of glycodendrimers thanks to the specific interactions with surface lectin receptors,^32,33^ providing receptor-mediated endocytosis.

In our previous studies, we determined the immunomodulatory properties of PPI dendrimers of the 4th generation surface-modified with maltose, cellobiose, and lactose moieties, demonstrating their ability to trigger inflammatory responses through the activation of NF-κB, AP-1, and NF-AT signaling pathways in myeloid cell-line models.^5,6^ While these studies were performed on glycodendrimers with full surface modification with sugar moieties (dense-shell), we have also collected a body of data on the bioactivity of partially maltose-modified (open-shell) PPI macromolecules developed predominantly as potential drug delivery agents.^19,20^ Here, we decided to use the availability of the remaining free amino groups to introduce secondary glyco-modification with mannose, one of the best-tested carbohydrates in terms of immunomodulation,^34^ as well as the most commonly used for modification of nanoparticles.^35^ The open-shell scaffold allowed us to attach mannose via a PEG chain to increase its potential accessibility for membrane receptors, in contrast to previous work on dense-shell glycodendrimers where sugar residues are located directly on the surface of the PPI dendrimer. We demonstrated the effective attachment of mannose via the PEG-based linkers in a manner that allows the direct observation of mannose-derived bioactivity and its comparison to bioactivity stemming from concurrently present maltose modification. Since we do not have data to speculate on the molecular identity of receptors responsible for the observed effects of mannose-modified glycodendrimers, the cellular fate (including potential internalization) of dendrimer molecules is not relevant to the present study since we do not identify the starting point of generated cellular signals that we investigate.

Mannose is a very important component of many microorganisms (including pathogenic ones) as a constituent of polysaccharides (mannans), glycoproteins, and glycolipids. Therefore, animals have evolved a number of systems for specific recognition of mannose-containing molecules, including membrane surface pattern recognition receptors (e.g., DC-SIGN or macrophage mannose receptor MRC1^36^) and soluble receptors (such as mannose-binding lectin, MBL^37^). These proteins are especially important within the tightly regulated immune system, where the ability to distinguish naturally occurring mammalian mannose-containing glycans from microbial mannose-containing glycans may be crucial to elicit pathogen response only when needed.^38^ This feature is complicated by the fact that foodborne mannans are a central source of mannose for the synthesis of mammalian glycans; so, mannose itself cannot be recognized unequivocally as a “danger signal” or PAMP (pathogen-associated molecular pattern). Thus, the diversity of cellular responses to mannose and mannose-containing biomacromolecules is a reflection of this multifacet mammalian relationship with mannose. Therefore, mannose has often been used as a bioactive modification of various nanoparticle types for immunomodulatory purposes, for example, chitosan nanoparticles with mannose moieties to enhance antitumor immunity^39^ or polymeric nanoparticles functionalized with mannose as adjuvants to produce anticancer vaccines.^40^

In this study, the central finding consists of the specific ability of mannose-containing glycodendrimers to stimulate the production of bioactive IL-8 by cells of the myeloid lineage. IL-8 is a member of the chemokine family, produced and secreted by a variety of normal and neoplastic human cell types, playing an important role in the chemotaxis of immune cells (primarily neutrophils) and induction of phagocytosis. Due to its potent pro-inflammatory properties, the expression of IL-8 is strictly regulated, being low or undetectable in unstimulated tissues. The best-characterized sequence in the IL-8 promoter contains binding sites for the NF-κB (required for its transcriptional activity), AP-1, and C-EBP/NF-IL-6 transcription factors (regulating transcription in a cell-dependent manner under certain pathophysiologic conditions). Expression of IL-8 can be induced by various stimulants, such as IL-1 and -6, tumor necrosis factor-α (TNF-α), interferon-γ (IFN-γ), lipopolysaccharides, phytohemagglutinin, phorbol myristate acetate, reactive oxygen species, or changes in cytosolic Ca^2+^ concentration. Cellular levels of IL-8 mRNA have been shown to be directly influenced both by its half-life and by transcriptional rates of the IL-8 gene. In addition to its well-described role in regulating inflammatory responses, IL-8 possesses distinct biological functions, including tumorigenic and proangiogenic properties. The latter is manifested through endothelial cell responses, including the enhancement of cell proliferation, chemotaxis, survival, and protease activation. IL-8 stimulates mRNA expression of matrix metalloproteinases (MMPs) −2 and −9, as well as gelatinase activity in endothelial cells. IL-8 has been shown to be critical for the development and progression of numerous malignancies, including gliomas and colorectal cancers. It has also been implicated in the pathology of cystic fibrosis and gastrointestinal inflammation.^9,41−43^

Considering multiple biological functions of IL-8, we decided to take a closer look at the mechanism of the modulation of its expression by glycodendrimers. Moreover, an even more interesting aspect of the study is the specificity of the immunomodulatory action resulting in increased production of one of the central myeloid-derived cytokine regulators by cells with different terminal differentiation potential (HL-60 with the ability to differentiate into multiple representatives of the myeloid lineage, THP-1 as a dedicated monocyte lineage cell type). Our study delved deeper into the molecular basics of the observed effect and provided a complex explanation that involves a mix of transcriptional and post-transcriptional effects, with the former predominating in HL-60 cells and the latter in THP-1 cells. Interestingly, a phenomenologically similar (though weaker) but mechanistically separate stimulation of IL-8 expression was also seen for the OS-PEG dendrimer which was a synthesis intermediate. We infer that this compound, which is slightly more toxic than the mannose-modified congener, may exert its stimulatory action on IL-8 biogenesis via stress response pathways unrelated to those shown to be involved in OS-PEG-Man activity.

Our data expand the knowledge on the mechanism of immunomodulation via the IL-8 axis but remains firmly within the known paradigms of influences on IL-8 expression. Thus, AP-1 transcriptional control of the *CXLC8* gene has been demonstrated in various cell types, including hepatocellular carcinoma^44^ and adrenocortical adenocarcinoma.^45^ Our study enhances this knowledge not only by adding mannosylated nanoparticles to the list of potential *CXLC8* inducers but also by demonstrating that this is not due to a direct effect on AP-1-regulating kinases, even though some of them have a central role in maintaining high constitutive IL-8 production in HL-60 cells. In this respect, mannose glycodendrimers are unlike, for example, TNF-α, which stimulates IL-8 production in connective tissue cells via activating the MAPK pathway,^46^ or interleukin-1β, which has a similar mechanism of action in gastric carcinoma.^47^ Conversely, in our study, the stimulatory effect of OS-PEG-Man on IL-8 production in THP-1 cells was shown to be mediated predominantly by a post-transcriptional mechanism of mRNA stabilization. A similar mode of action was demonstrated for p38 and ERK in lung epithelial cells.^48^ We show this mechanism to be particularly efficient as IL-8 production, which is negligible in THP-1 cells, reverts to a level comparable with HL-60 cells upon OS-PEG-Man treatment. A similar phenomenon was described in artificially differentiated THP-1 cells.^49^ The discovery of this complex regulatory mechanism in myeloid cells at different levels of differentiation confirms the notion that mannose-containing compounds, especially well-characterized and monodisperse nanoparticles, can be efficiently used for immunomodulation at basal nodes of immune regulatory networks where myeloid cells take center stage.

Our mechanistic results pave the way to potential applied research in order to utilize in practice the capability of mannose glycodendrimers with a novel architecture to influence important cellular pathways in myeloid cells. Specifically, enhanced production of IL-8 by myeloid cells is of obvious advantage in topical applications at acute infection sites (infected wounds, mucosal infection foci), including in immunosuppressed individuals, since it is a granulocyte-attracting chemokine that would enhance pathogen killing and wound healing. Thus, its action would parallel existing immunomodulatory preparations with multiple clinical indications, such as imidazoquinolinamine compounds.^50^ More generally, mRNA stabilization as a mechanism of cytokine upregulation (especially with cell-type-restricted specificity, as demonstrated in this study) is a novel feature of functionalized dendrimers, closely linked to their high biocompatibility and capacity for uptake into cells.^51^ It should be further pursued for other peptides important for immune cell communication and function. In the special case of IL-8, our study is an important contribution to the body of knowledge on its modes of regulation. While IL-8 mRNA stability has been previously demonstrated as an important control point for its abundance,^52,53^ we were able to use it for targeted exogenous induction of IL-8 for immunomodulatory purposes.

Immunomodulation is just one aspect of the excellent applicability of dendrimers for pharmacodynamic aims with direct biological effects mediated both by their inherent properties and by functionalization. It is important to note that often the same molecular mechanisms can underlie physiological actions of nanoparticles (which can be exploited for pharmaceutics) and deleterious nanotoxicological effects. Thus, basic studies of subcellular pathways for validating their interaction profile with different nanoparticles are fundamental for both fields. Dendrimers are a special case due to their uniformity, modular structure, ease of design, and characterization,^1,54^ yielding a general simplicity that is especially useful for proof-of-concept studies. While the present article deals with the effect of mannose functionalization of glycodendrimers on the expression of a specific cytokine in two cell types, it provides a generic conceptual framework for further studies in this field, which are currently underway in our group and others.

## Conclusions

Our proof-of-concept study resulted in two main outcomes: discovery of the ability of mannose-modified glycodendrimers to induce the expression of IL-8 in myeloid cells and identification of the molecular mechanism involved in this phenomenon. Moreover, this mechanism depended on the cellular context, with control of mRNA degradation most important in the THP-1 cell line (where initial IL-8 expression was low) and transcriptional induction effective in the HL-60 cell line (with higher initial IL-8 level). The dendrimers we used were designed for directed immunomodulation by attachment of mannose so as to sterically facilitate receptor interactions. The success of this approach with innovative pathways of synthetic design guided by bioactivity requirements opens new possibilities for designing immunomodulatory nanoparticles.

## Supporting Information Available

Description of synthesis and characterization of glycodendrimers applied in the presented research (PDF)
