# Supplementary material for: Nanoparticles for Directed Immunomodulation: Mannose-Functionalized Glycodendrimers Induce Interleukin-8 in Myeloid Cell Lines
Source: Biomacromolecules. 2021 Jul 21;22(8):3396–407. doi: 10.1021/acs.biomac.1c00476 (PMC8382243; doi:10.1021/acs.biomac.1c00476)
Supplement: Supplementary file 1 — bm1c00476_si_001.pdf [file bm1c00476_si_001.pdf]

Supporting Information

Nanoparticles for directed immunomodulation: Mannose-functionalized glycodendrimers induce interleukin-8 in myeloid cell lines

Izabela Jatczak-Pawlik[1,2,3], Michał Gorzkiewicz[3], Maciej Studzian[4], Robin Zinke[5], Dietmar Appelhans[5], Barbara Klajnert-Maculewicz[3], Łukasz Pułaski[4,6]

1. Department of Hypertension, Chair of Nephrology and Hypertension, Medical University of Lodz, 281/289 Rzgowska St., 93-338 Lodz, Poland
2. Polish Mother's Memorial Hospital Research Institute (PMMHRI), 281/289 Rzgowska St., 93-338 Lodz, Poland
3. Department of General Biophysics, Faculty of Biology and Environmental Protection, University of Lodz, 141/143 Pomorska St., 90-236 Lodz, Poland
4. Department of Molecular Biophysics, Faculty of Biology and Environmental Protection, University of Lodz, 141/143 Pomorska St., 90-236 Lodz, Poland
5. Leibniz Institute of Polymer Research Dresden, Hohe Straße 6, 01069 Dresden, Germany
6. Laboratory of Transcriptional Regulation, Institute of Medical Biology PAS, 106 Lodowa St., 93-232 Lodz, Poland

SYNTHESIS OF DENDRIMERS

Materials

(Benzotriazol-1-yloxy)tris(dimethylamino)phosphonium hexafluorophosphate (BOP), M_w = 442.28 g/mol); 4th generation poly(propyleneimine) dendrimer (M_w = 7162.96 g/mol) (**G4**),# (maltose monohydrate, 360.31 g/mol), alpha-tert-butyloxycarbonylamino-omega-diglycolic acid octa(ethylene glycol) (PEG_8, 584.67 g/mol), dimethyl sulfoxide (DMSO), Borane*pyridine complex (BH_3*Pyr, 8 M), HCl/dioxane solution (4M, 36.46 g/mol); (1R,8S,9s)-bicyclo[6.1.0]non-4-yn-9-ylmethyl succinimidyl carbonate (COD, 291.3 g/mol); azido-modified α-D-mannose (263.25 g/mol; Man-N_3).

Comparing polyamine dendrimer of different compositions we followed the suggestion by Tomalia and Rookmaker[1]. As a result, the commercially available 5th generation dendrimer DAB-Am64 is described as 4th generation PPI dendrimer here.

Characterization techniques. **^{1}H NMR Spectroscopy** experiments were carried out using a Bruker Avance III 500 NMR spectrometer operating at 500.13 MHz. Either DMSO-d_6 or deuterium oxide (D_2O) was used as solvent. Chemical shifts were referenced on solvent peaks (DMSO-d_6, δ = 2.50 ppm, D_2O, δ = 4.75 ppm). **Matrix-Assisted Laser Desorption/Ionization-Time of Flight Mass Spectrometry (MALDI-TOF MS)** investigations were performed on a Bruker Autoflex Speed TOF/TOF in reflector or linear modes, respectively, and positive polarity by pulsed smart beam laser (modified Nd:YAG laser). The ion acceleration voltage was set to 20 k. For the sample preparation, the substances were mixed with 2,5-dihydroxy benzoic acid as matrix, both dissolved in millipore water.

Synthesis of Man-N_3

Man-N_3 was synthesized followed a synthetic protocol published[2]. **Man-N_3** was characterized by ^{1}H NMR in D_2O.

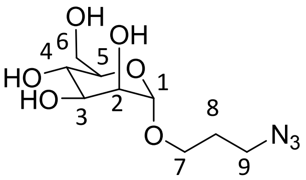

^{1}H NMR (D_2O): δ = 1.95 (m, 8), 3.47 (m, 9), 3.62 (q, 7A), 3.66 (m, 5), 3.67 (m, 4), 3.79 (d, 6A), 3.83 (m, 3), 3.84 (q, 7B), 3.92 (d, 6B), 3.98 (dd, 2), 4.89 ppm (d, 1).

Synthesis of PPI-PEG

Anhydrous DMSO (40 mL) was dried and degassed for around 1 h under high vacuum. In a first reaction flask PPI dendrimer G4 was dissolved in 2/3 (of 40 mL) of anhydrous DMSO and then triethylamine (Et_3N) was added to the reaction mixture under argon protection atmosphere. In a second reaction flask alpha-tert-butyloxycarbonylamino-omega-diglycolic acid octa(ethylene glycol) and BOP were dissolved in 1/3 (of 40 ml) of anhydrous DMSO. Then, the resulting reaction mixture was stirred for 1 h under argon atmosphere. PPI dendrimer solution was slowly added to the activated PEG_8 solution under protection atmosphere. This reaction mixture was then stirred for 3 days at room temperature under argon atmosphere and was then dialyzed for 2 days in water (membrane tube with MWCO 2000 g/mol). After the freeze drying a viscous liquid was obtained. Used quantities for **PPI-PEG** are presented in Table S1.

Table S1. Used educts for the synthesis of **PPI-PEG**: PPI-PEG-I used for **PPI-PEG-Man** and PPI-PEG-II used for **PPI-PEG-COD**.

PPI-PEG	G4	PEG_8	BOP	Et_3N	DMSO	Yield
	eq [mol]	eq [mol]	eq [mol]	[mL]	[mL]	[g] [%]

	[mg]	[mg]	[mg]			
**PPI-PEG-I**	1.0<br>1.46×10^{-4}<br>1046	6.0<br>8.76×10^{-4}<br>512	6.5<br>9.49×10^{-4}<br>420	1.0	40	1.510<br>98.1
**PPI-PEG-II**	1.0<br>1.505×10^{-4}<br>1078	6.0<br>9.030×10^{-4}<br>528	6.5<br>9.782×10^{-4}<br>433	1.0	40	1.574<br>95.1

^{1}H NMR (D_2O): δ = 1.42 (12´´), 1.55-1.8 (a, d, g, j, m, p), 2.35-2.7 (b, c, e, f, h, i, k, l, n, o), 2.75-2.9 (q), 3.01 (not assigned), 3.25 (x, 9´´), 3.45 (5´´), 3.57 (8´´), 3.64 (6´´), 3.68 (7´´), 4.11 and 4.12 ppm (2´´, 3´´) (**Figure S2**).

^{13}C NMR (D_2O): δ = 24-29 (a, d, g, j, m, p), 30.7 (12´´), 40.3 (x), 41.4 (q, 5´´), 42.3 (not assigned), 42.6 (9´´), 52-57 (b, c, e, f, h, i, k, l, n, o), 71.6 (6´´), 72.3 (8´´), 72.5 (7´´), 72.8 and 72.9 (2´´ and 3´´), 83.8 (11´´), 161.0 (10´´), 167.4 (not assigned), 174.3 and 174.5 ppm (1´´, 4´´) (**Figure S2**).

Synthesis of maltosylated PPI-PEG-I (PPI-Mal-PEG-I) and PPI-PEG-II (PPI-Mal-PEG-II)

PPI-PEG (theoretical M_w = 10567 g/mol) was stirred for one hour at 50 °C to dissolve it in sodium borate buffer (0.1 M; Na borate). After that maltose was added to the reaction mixture which was stirred for one hour at 50 °C to completely dissolve the solid maltose in the reaction mixture. Borane*pyridine complex (BH_3*Pyr) was lastly added to the reaction mixture, and this mixture was stirred for 7 days at 50 °C under reflux. Then the crude product was dialyzed for 4 days in water (membrane tube with MWCO of 2000 g/mol) and after freeze drying process a white solid was obtained. Used quantities for **PPI-Mal-PEG-I** and **PPI-Mal-PEG-II** are presented in Table S2.

Table S2. Used educts for the synthesis of **PPI-Mal-PEG**: PPI-Mal-PEG-I used for **PPI-PEG-Man** and PPI-Mal-PEG-II used for **PPI-PEG-COD**.

**PPI-Mal-PEG**	**PPI-PEG**		**Maltose**	**BH_3*Pyr**	**Na borate**	**Yield**
		eq<br>[mol]<br>[mg]	eq<br>[mol]<br>[g]	eq<br>[mol]<br>[mL]	[mL]	[g]<br>[%]
**PEG-Mal-PEG-I**	**PPI-PEG-I**	1.0<br>9.5×10^{-6}<br>100	64<br>6.1×10^{-4}<br>0.220	64<br>6.1×10^{-4}<br>0.08	20	0.170<br>85.2
**PEG-Mal-PEG-II**	**PPI-PEG-II**	1.0<br>3.520×10^{-5}<br>372	58<br>2.042×10^{-3}<br>0.736	58<br>2.042×10^{-3}<br>0.3	25	0.729<br>90.8

^{1}H NMR (D_2O): δ = 1.43 (8´´), 1.55-2.1 (a, d, g, j, m, p), 2.25-3.2 (b, c, e, f, h, i, k, l, n, o, q, 1´), 3.25 (x, 7´´), 3.4-4.5 (2-6, 2´-6´, 1´´-6´´), 5.05-5.35 ppm (1) (**Figure S3**).

Deprotecting of PPI-Mal-PEG for synthesizing PPI-Mal-PEG-NH$_2$

Polymer was dissolved in deionized water. HCl/dioxane solution (4M, 1.05 g/mL) was added dropwise to the reaction mixture under stirring. The resulting reaction mixture was then stirred for 24h at room temperature. This mixture was dialyzed for 2 days in deionized water (membrane tube with MWCO of 2000 g/mol). After the freeze drying a white solid was obtained. Used quantities for **PPI-Mal-PEG-NH$_2$** are presented in Table S3.

Table S3. Used educts for the synthesis of **PPI-Mal-PEG-NH$_2$**: PPI-Mal-PEG-I-NH$_2$ used for **PPI-PEG-Man** and PPI-Mal-PEG-II-NH$_2$ used for **PPI-PEG-COD**

**PPI-Mal-PEG-NH$_2$**	**PPI-Mal-PEG**		**HCl /dioxane**	**Water**	**Yield**
		Eq<br>[mol]<br>[mg]	eq<br>[mol]<br>[mL]	[mL]	[mg]<br>[%]
**PEG-Mal-PEG-I-NH$_2$**	PEG-Mal-PEG-I	1.0<br>7.14×10^{-6}<br>150	10000<br>0.0714<br>2.5	5	150<br>105.0
**PEG-Mal-PEG-II-NH2**	PEG-Mal-PEG-II	1.0<br>1.715×10^{-5}<br>391	10000<br>0.171<br>5.9	20	387<br>100.7

^{1}H NMR (D_2O): δ = 1.6-2.0 (a, d, g, j, m, p), 2.45-3.30 (b, c, e, f, h, i, k, l, n, o, q, x, 1´, 7´´), 3.3-4.2 (2-6, 2´-6´, 1´´-6´´), 5.19 ppm (1) (**Figure S4**).

Conversion of PPI-Mal-PEG-NH$_2$ with COD (1)

Anhydrous DMSO (40 mL) was dried and degassed for around 1h under high vacuum. In a first reaction flask **PPI-Mal-PEG-NH$_2$** was dissolved in 2/3 (of 40 mL) of pretreated DMSO and triethylamine (Et_3N) was added. In a second reaction flask (1R,8S,9s)-bicyclo[6.1.0]non-4-yn-9-ylmethyl succinimidyl carbonate (COD) was dissolved in 1/3 (of 40 mL) of preteated DMSO and then stirred for 1 h under argon protection atmosphere. PPI dendrimer solution was slowly added to the COD-containing solution under argon protection atmosphere. This reaction mixture reacted for 3 days at room temperature under argon atmosphere and protected from light. Then the resulting crude product was dialyzed for 2 days in deionized water (membrane tube with MWCO of 2000 g/mol). After freeze drying process a white solid was obtained. Used quantities for converted **PPI-Mal-PEG-NH$_2$** with COD are presented in Table S4.

Table S4. Used educts for the conversion of **PPI-Mal-PEG-NH$_2$** with COD: PPI-Mal-PEG-I-COD further used for **PPI-PEG-Man** and PPI-Mal-PEG-II-NH$_2$ used for **PPI-PEG-COD**

**PPI dendrimer**	**PEG-Mal-PEG-NH$_2$**		**COD**	**NEt$_3$**	**DMSO**	**Yield**
		eq<br>[mol]<br>[mg]	eq<br>[mol]<br>[mg]	[mL]	[mL]	[mg]<br>[%]
**PEG-Mal-PEG-I-COD**	PEG-Mal-PEG-I-NH$_2$	1.0<br>5.00×10^{-6}<br>100	24.0<br>1.20×10^{-4}<br>35.0	0.5	15	115<br>70.2
**PPI-PEG-COD**	PPI-Mal-PEG-II-NH$_2$	1.0<br>1.539×10^{-5}<br>351	24.0<br>3.695×10^{-4}<br>107.7	1	30	352<br>77.8

^{1}H NMR (D_2O): δ = 0.9-1.15 (9´´, 10´´), 1.6-2.0 (a, d, g, j, m, p, 11´´, 12´´), 2.45-3.30 (b, c, e, f, h, i, k, l, n, o, q, x, 1´, 7´´), 3.3-4.2 (2-6, 2´-6´, 1´´-6´´, 8´´), 5.19 ppm (1) (**Figure S5 + S6**).

Synthesis of PPI-PEG-Man

PPI-Mal-PEG-I-COD was dissolved in PBS buffer (0.01M), and azido-modified mannose (2) was dissolved in methanol. Both solutions were degassed with 3 freeze-pump-cycles followed by a 1h stirring at room temperature under argon atmosphere. Then adding the PPI dendrimer solution to 2-containing solution and let stirred the resulting reaction for 3 days at 35 °C under argon and light protection. The crude product was then dialyzed in deionized water with steady water exchange for 2 days (membrane tube with MWCO of 2000 g/mol) and after a freeze drying a light yellow solid was obtained. Used quantities for the synthesis of **PPI-PEG-Man** are presented in Table S5.

Table S5. Used educts for the synthesis of **PPI-PEG-Man**

**Compound**	**Precursor**		**2**	**PBS buffer**	**Methanol**	**Yield**
		Eq<br>[mol]<br>[mg]	eq<br>[mol]<br>[mg]	[mL]	[mL]	[mg]<br>[%]
**PPI-PEG-Man**	PPI-Mal-PEG-II-NH$_2$	1.0<br>3.51×10^{-6}<br>100	10.0<br>3.51×10^{-5}<br>9.2	10	2	90<br>95.0

^1H NMR (D_2O): δ = 1.25-2.0 (a, d, g, j, m, p, 9´´-11´´), 2.1-3.3 (b, c, e, f, h, i, k, l, n, o, q, x, 1´, 7´´, 12´´, 14´´), 3.3-4.2 (2-6, 2´-6´, 1´´-6´´, 8´´, 15´´, 17´´-21´´), 4.75-5.0 (16´´, overlapped by H_2O), 5.12 ppm (1) (**Figure S7**).

Synthesis of PPI-Mal

G4 was stirred for 1h at 50 °C prior taken up in sodium borate buffer (0.1 M; Na borate). After the addition of maltose the reaction mixture was stirred for 1h at 50 °C again. Borane*pyridine complex (BH_3*Pyr) was finally added to the reaction which was subsequently stirred for 7 days at 50 °C under reflux. The desired crude product was dialyzed for 4 days in deionized water with steady water exchange (membrane tube with MWCO of 2000 g/mol) and after a freeze drying step a white solid was obtained. Used quantities for the synthesis of **PPI-Mal** are presented in Table S6.

Molecular weight and composition of poly(propyleneimine) glycodendrimers are presented in Table S7.

Table S6. Used educts for the synthesis of **PPI-PEG-Man**

**Compound**	**Precursor**		**Maltose**	**BH_3*Pyr**	**Na borate**	**Yield**
		eq<br>[mol]<br>[mg]	eq<br>[mol]<br>[g]	eq<br>[mol]<br>[mL]	[mL]	[g]<br>[%]
**PPI-Mal**	**G4**	1.0<br>5.58×10^{-5}<br>400	64<br>3.57×10^{-3}<br>1.29	128<br>7.14×10^{-3}<br>0.9	25	1.088<br>92.8

^1H NMR (D_2O): δ = 1.5-2.1 (a, d, g, j, m, p), 2.25-3.2 (b, c, e, f, h, i, k, l, n, o, q, 1´), 3.4-4.5 (2-6, 2´-6´), 5.05-5.35 ppm (1) (**Figure S8**).

Table S7. Molecular weight and molecular composition of poly(propylene imine) glycodendrimers for biological assays.

**Dendrimer**	**MALDI-TOF MS**	**Number of**		
	M_w (g/mol)	maltose	PEG_8	Boc
**OS**<br>PPI-Mal	21,000	43[a, b]	-	-
PPI-PEG-II	11,000	-	6.2,[a] 6.5[b]	6.2,[a] 6.5[b]
PPI-Mal-PEG-II	22,800	42,[a] 36[b]	6.0,[a]	6.0,[a]
PPI-Mal-PEG-II-NH_2	22,400	-	-	degraded[a, b, e]

**OS-PEG**<br>PPI-PEG-COD[d]	29,400	-	-	-
PPI-PEG-I	9,500	-	6.2,[a] 4[b,c]	6.2[a]
PPI-Mal-PEG-I	21,000	46,[a] 36[b]	5.9[a]	5.9[a]
PPI-Mal-PEG-I-NH_2	20,000	-	-	degraded[a, b, e]
PPI-PEG-I-COD[d]	28,500	-	-	-
**OS-PEG-Man**<br>(PPI-PEG-Man[d])	27,000	-	-	-

[a]Determined by ^{1}H NMR. [b]Determined by MALDI-TOF MS. [c]Degradation of PPI dendrimer during MALDI-TOF MS. [d]Determination for the number of COD by MALDI-TOF MS is hampered due to potential encapsulation of matrix material, showing to high number of COD and molecular weight (M_w). Therefore, matrix encapsulation by PPI-PEG-Man is assumed due to high M_w. [e]Indication of degraded PEG-spacer and/or part of PPI scaffold in PPI-Mal-PEG-I/II-NH_2 during MALDI-TOF MS.

^{1}H NMR characterization of different dendritic structures used in this study

^{1}H NMR spectrum of PPI-PEG

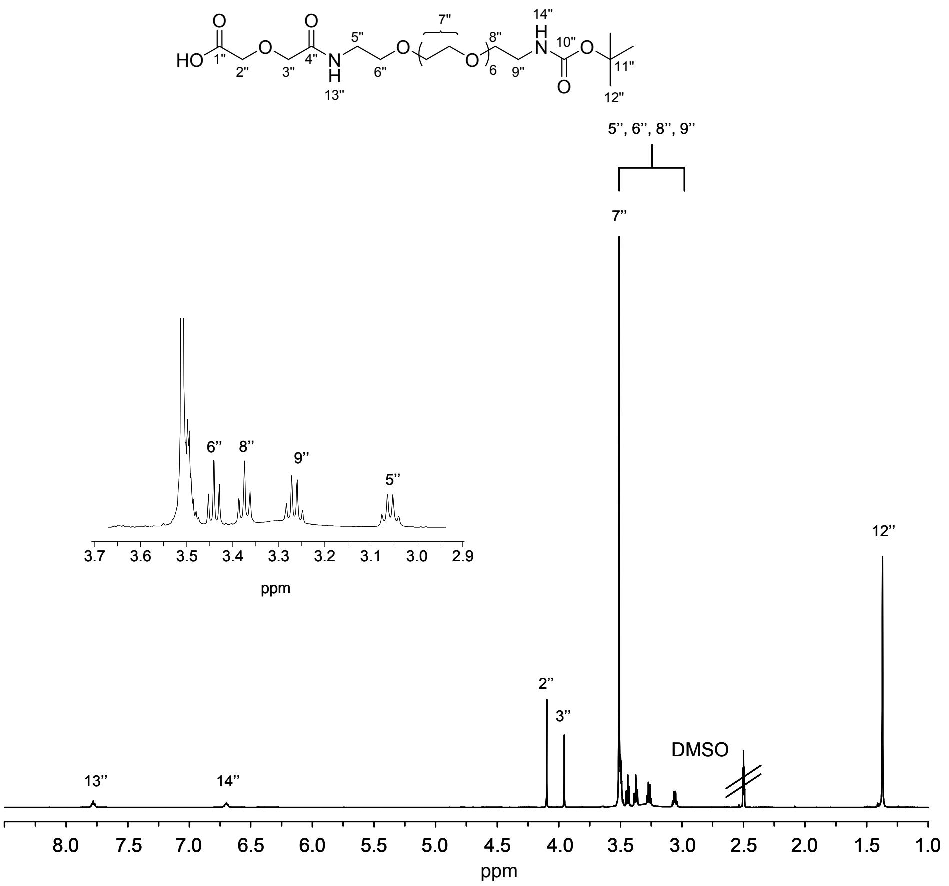

Figure S1. ^{1}H NMR spectrum of NH-Boc-PEG$_8$-CO$_2$H, presented in Figure 1 of paper, in DMSO-d6. This precursor was used to synthesize PPI glycodendrimers OS-PEG and OS-PEG-Man.

Determination of coupled PEG-Spacer on OS-PEG and OS-PEG-Man

The ^{1}H NMR spectrum of PPI-PEG (**Figure S2**) can be used to determine the level of coupled PEG units (NH-Boc-PEG$_8$-CO$_2$H) on the surface of 4th generation (PPI-G4) scaffold: The integral value of the four protons of 2´´ and 3´´ (~ 4.2 ppm) gives the intensity of one proton of the attached PEG units. The integral value of the 1.25 – 2.0 ppm region gives the intensity of protons a, d, g, j, m, p of the PPI-G4 scaffold (signal assignment of PPI scaffold is presented in **Figure S8**) and 12´´. This large region was used to avoid integration errors due to signal overlap. This integral value is corrected by the integral of 12´´ calculated from the intensity of 2´´ and 3´´. The corrected integral represents 252 protons of PPI-G4 and thus the intensity of one proton of the PPI-G4 can be calculated. PPI-G4 contains 64 amino end groups. Relating the intensity of 64 amino protons of PPI-G4 to the intensity of the PEG unit gives the degree of modification. This method was used to calculate 6-6.2 PEG units per PPI-G4 macromolecule (Figure S11). Thus, 6 PEG units per PPI-G4 macromolecule were assumed for calculating the attached maltose units for glycodendrimers OS-

PEG and OS-PEG-Man (**Table S1**). In ^{1}H NMR spectrum of PPI-Mal-PEG the *tert.* butyl of NH-Boc group as broad signal (8′′) is still visible below 1.5 ppm (**Figure S3**) after reductive amination of PPI-PEG (precursor of PPI-Mal-PEG). Assignment of a-q for PPI-G4 scaffold is adapted from former publication (Klajnert et al., *Chem. Eur. J.* **2008**, *14*, 7030).

^{1}H and ^{13}C NMR spectra of PPI-PEG

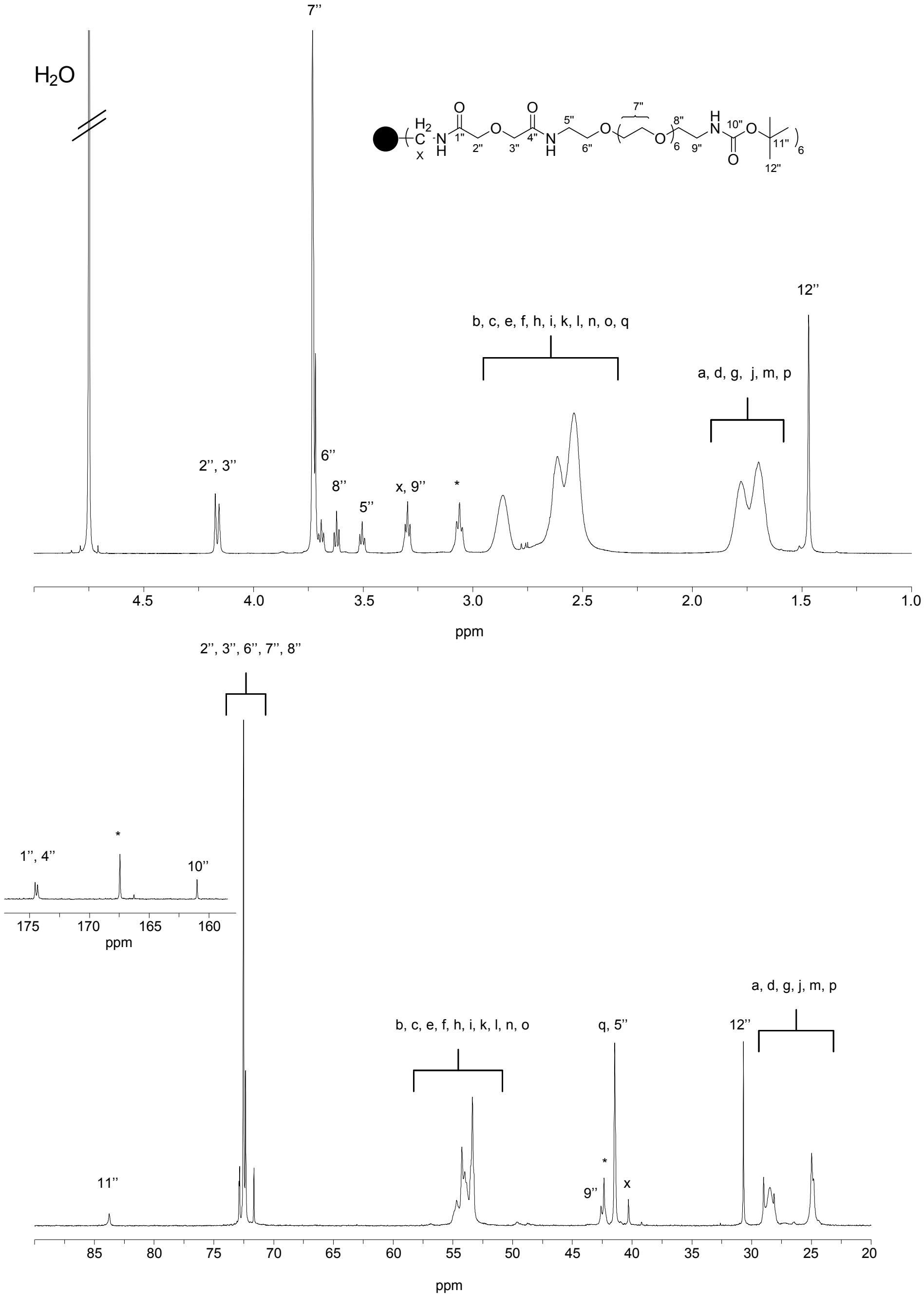

Figure S2. ^{1}H and ^{13}C NMR of PPI-PEG in D_2O. This macromolecule was used as a precursor for synthesizing PPI glycodendrimers PPI-PEG-NH_2 and other PEG-containing PPI dendrimers, OS-PEG

and OS-PEG-Man. Assignment of a-q for PPI-G4 scaffold is adapted from former publication (Klajnert et al., *Chem. Eur. J.* **2008**, *14*, 7030.)

^{1}H NMR characterization of OS-PEG

^{1}H NMR characterization of PPI-Mal-PEG

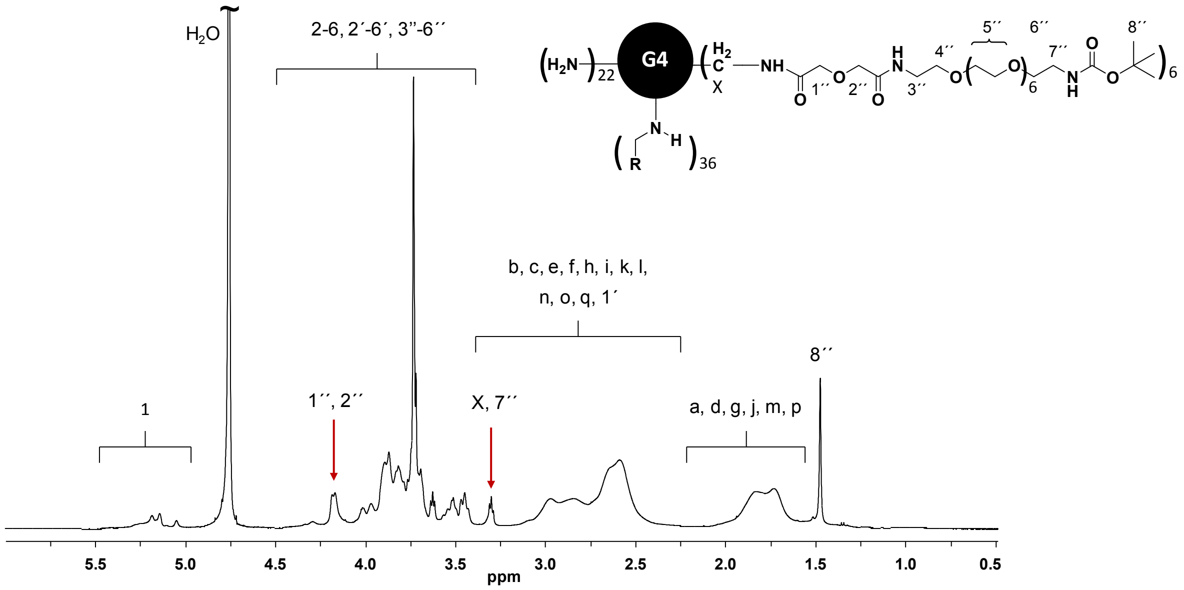

Figure S3. ^{1}H NMR of PPI-Mal-PEG in D_2O. This macromolecule was used as a precursor for synthesizing PPI glycodendrimers PPI-PEG-NH_2 and other PEG-containing PPI dendrimers. Assignment of a-q for PPI-G4 scaffold is adapted from former publication (Klajnert et al., *Chem. Eur. J.* **2008**, *14*, 7030).

^{1}H NMR characterization of PPI-Mal-PEG-NH_2

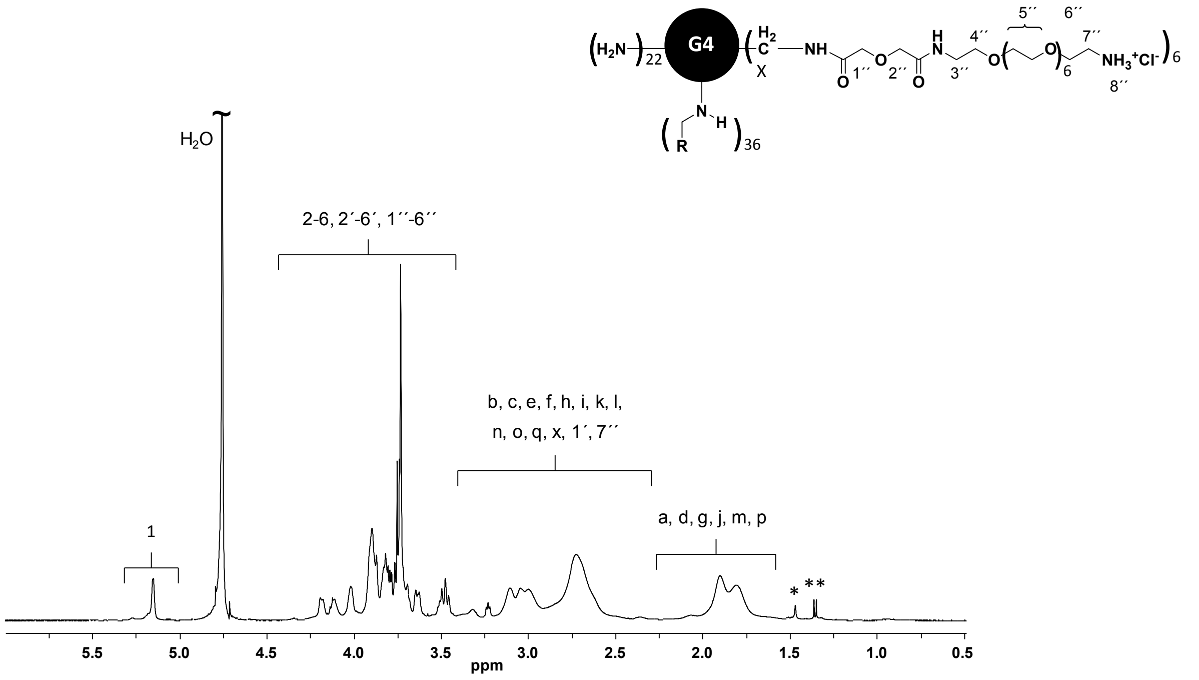

Figure S4. ^{1}H NMR of PPI-Mal-PEG-NH_2 in D_2O. This macromolecule was used as a precursor for synthesizing PPI glycodendrimers OS-PEG and OS-PEG-Man. Assignment of a-q for PPI-G4 scaffold is adapted from former publication (Klajnert et al., *Chem. Eur. J.* **2008**, *14*, 7030). Signal with * can be not identified, probably very less amount of NH-BOC of PPI-PEG. Signal with ** cannot be identified. Ammonium groups (NH_3^+, 8´´) can be elsewhere, but also protonated NH_2 groups of PPI-Mal-PEG-NH_2 can be elsewhere available.

^{1}H NMR characterization of OS-PEG

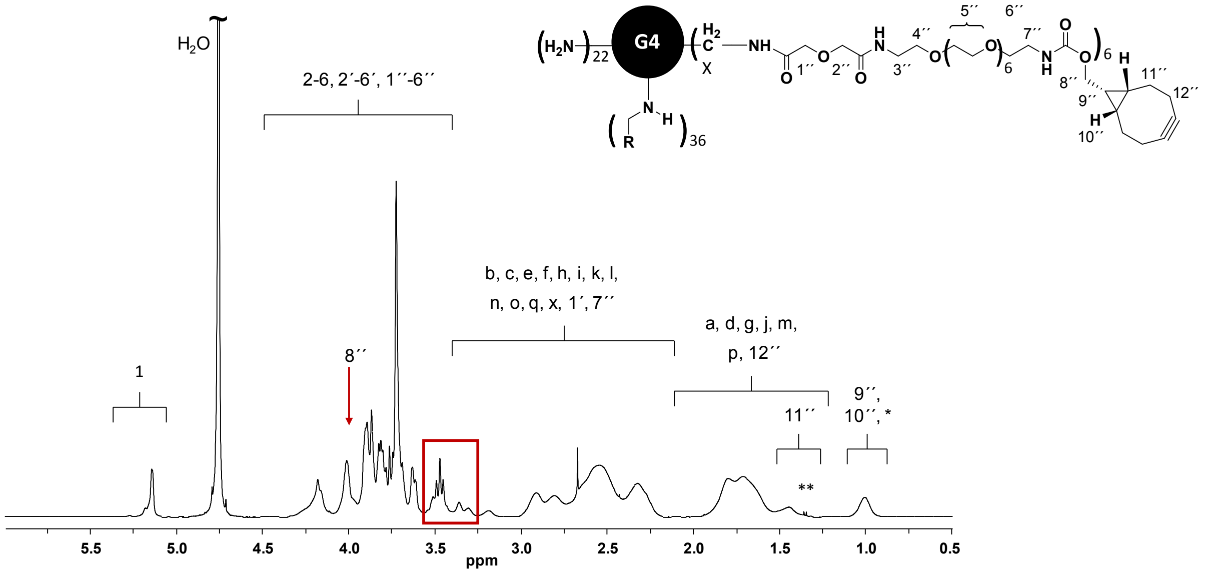

Figure S5. ^{1}H NMR of **OS-PEG** in D_2O. Assignment of a-q for PPI-G4 scaffold is adapted from former publication (Klajnert et al., *Chem. Eur. J.* **2008**, *14*, 7030). Explanation of signal with * and ** will be given below.

^{1}H NMR characterization of OS-PEG-Man

^{1}H NMR characterization of OS-PEG

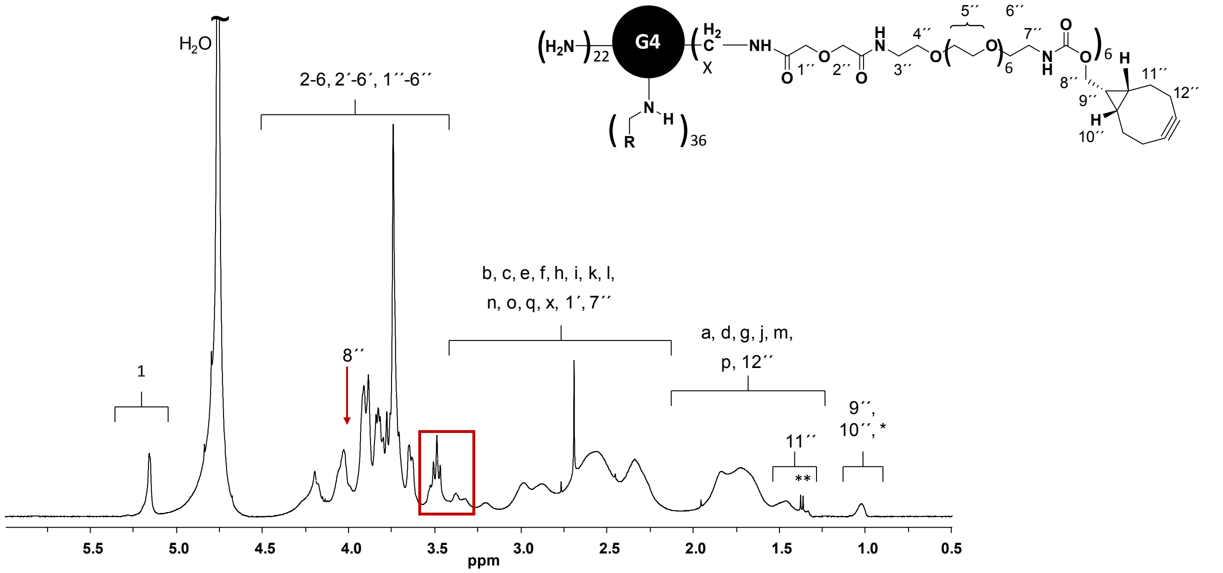

Figure S6. ^{1}H NMR of **OS-PEG** in D_2O. Assignment of a-q for PPI-G4 scaffold is adapted from former publication (Klajnert et al., *Chem. Eur. J.* **2008**, *14*, 7030). Explanation of signal with * and ** will be given below.

^{1}H NMR characterization of OS-PEG

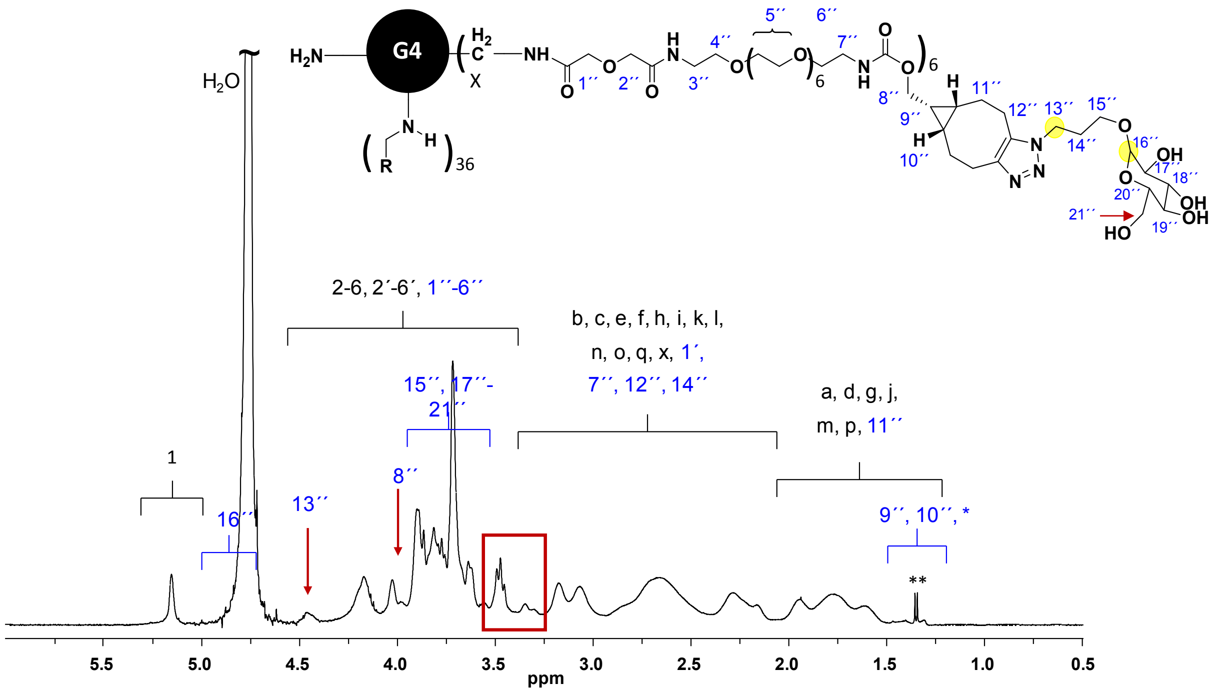

Figure S7. ^{1}H NMR of **OS-PEG-Man** in D_2O. Assignment of a-q for PPI-G4 scaffold is adapted from former publication (Klajnert et al., *Chem. Eur. J.* **2008**, *14*, 7030). Explanation of signal with * and ** will be given below.

Analysis of ^{1}H NMR spectra of OS-PEG and OS-PEG-Man

^{1}H NMR spectrum of PPI-Mal-PEG-NH_2 (**Figure S4**) outlines the required deprotection of *tert.* butyl group of NH-Boc group of PPI-Mal-PEG (**Figure S3**). Typical broad singulet of *tert.* butyl group (8´´; slightly below 1.5 ppm) in PPI-Mal-PEG is almost disappeared in ^{1}H NMR spectrum of PPI-Mal-PEG-NH_2. There could be a low content of residual signal of 8´´ for *tert.* butyl group of NH-Boc. This weak signal (slightly below 1.5 ppm) could be another signal which completely disappeared when deprotonated PPI-Mal-PEG-NH_2 is converted with COD derivative (**Figure 1**) to realize OS-PEG (**Figure S5**) and OS-PEG (**Figure S6**) for synthesizing OS-PEG-Man.

In both ^{1}H NMR spectra of OS-PEG (**Figure S5** and **S6**) we follow the signal assignment of attached COD derivative published in following reference: Biomacromolecules **2020**, *21*, 199-213. Signals, 7´´ and 8´´-12´´, belong to the COD derivative in OS-PEG. Signals, 8´´, 11´´ and 12´´, are overlapped by signal of maltose units for 8´´ and PPI scaffold for 11´´ and 12´´. Proton signals, 9´´ and 10´´, belong to the cyclopropane ring in COD derivative and are visible between 1.0 – 1.2 (**Figure S5** + **S6**), once coupled on a singular PEG spacer (Biomacromolecules **2020**, *21*, 199-213). Due to its hydrophobicity

of cyclopropane ring in the COD derivative, we assume only broadening of the proton signals and low intensity of its protons here. It is true, one can see a broad ^{1}H NMR signal (e.g. **Figure S5**) which cannot belong to proton signals of cyclopropane ring in D_2O, but presenting other structure units in OS-PEG (*). This broad signal (1-1.2 ppm) completely disappears when OS-PEG is converted with azido-mannose derivative to OS-PEG-Man (**Figure S7**). Proton 11´´ of COD derivatives is smoothly available on singular PEG chain (1.25-1.5 ppm), but not in case of OS-PEG, while another broad ^{1}H NMR signal appears in this region of OS-PEG (1.25-1.5 ppm) which is not present in the precursor, PPI-Mal-PEG-NH_2 (**Figure S4**). Triple bond is also not visible in FT-IR and Raman spectra, as known from terminal yne units. Beside this, reproducible ^{1}H NMR signal patterns are still visible in both OS-PEG, e.g. in red frame (**Figure S5** and **S6**).

In the ^{1}H NMR spectrum of OS-PEG-Man (**Figure S7**) one can recognize the disappearance of broad signals (1.0-1.15 ppm for 9´´ and 10´´ and 1.26-1.5 ppm for 11´´) and the appearance of significant broad ^{1}H NMR signal below 4.5 ppm (13´´) and from 2.1 to 3.25 ppm (12´´) and less intensive signals between 1.25 and 1.5 ppm. Beside the repeating appearance of significant ^{1}H NMR signal pattern (e.g. in red frame and anomeric proton of reductive glucose unit in the attached maltose unit on PPI scaffold), undoubtedly belonging to the OS-PEG scaffold, new important ^{1}H NMR signal appears slightly below 4.5 ppm, identified as CH_2 group (13´´) next to triazole ring (**Figure S7**). 13´´ was predicted as ^{1}H NMR signal, while anomeric proton (16´´) of mannose is preferentially overlapped by the water signal (4.75-5.0 ppm). Proton 16´´ of OS-PEG-Man is clearly assignable in ^{1}H NMR spectrum of 1-azidopropyl modified mannose (4.89 ppm) in D_2O (**Figure S10**).

Results of MALDI-TOS MS confirm the successful modification of COD derivative and azidopropyl mannose starting from PEG-Mal-PEG-NH_2 in both cases for OS-PEG and OS-PEG-Man (**Table S7**), leading to increasing molar masses. Precise molar mass of OS-PEG and OS-PEG-Man is completely/partly hampered through postulated matrix material encapsulation.

^{1}H NMR spectrum of PPI-Mal

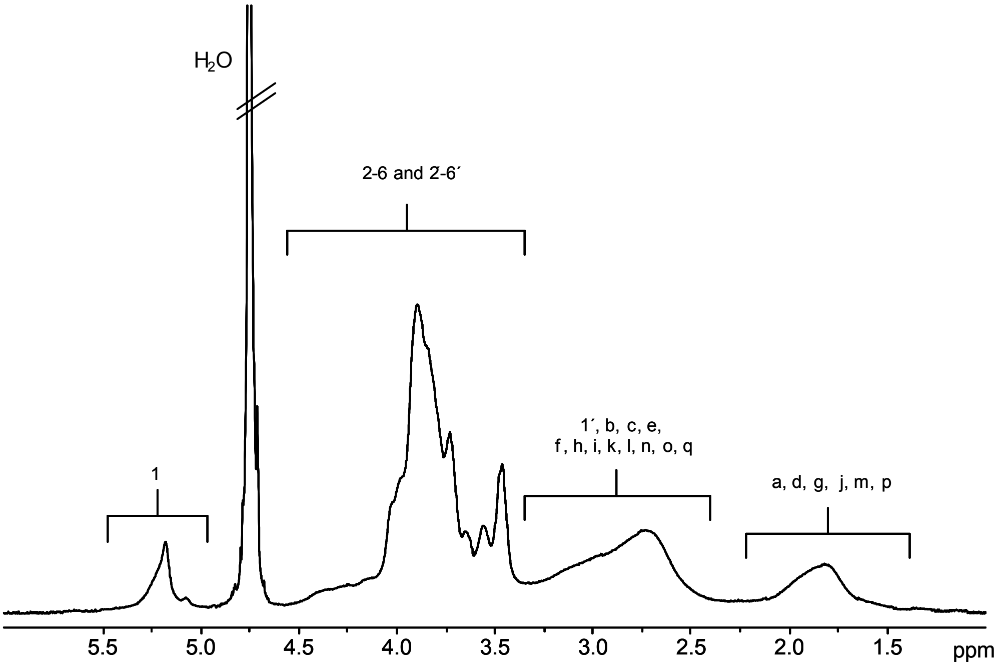

Figure S8. ^{1}H NMR spectra of **OS** (PPI-Mal based on PPI-G4 scaffold) in D_2O.

Determination of ^{1}H NMR signals of PPI dendrimer scaffold

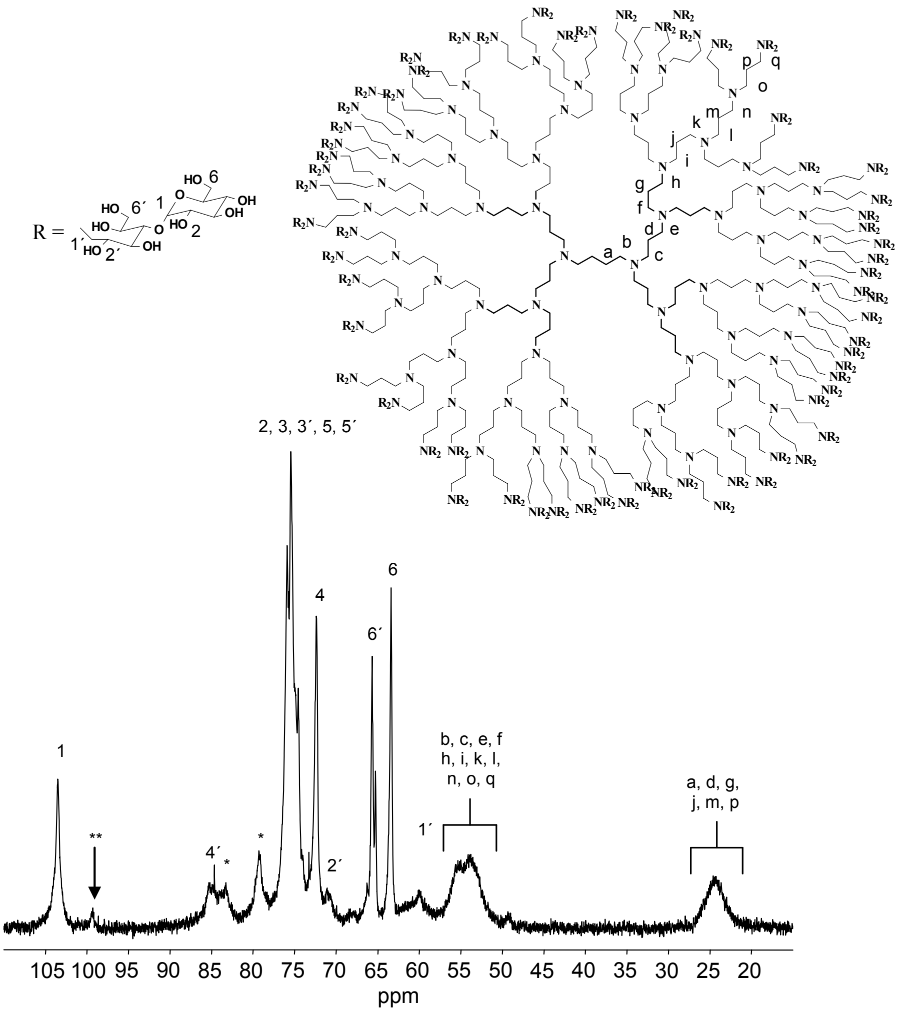

Figure S9. ^{13}C NMR spectrum of maltosylated PPI-G4 with dense shell in D_2O (* indicates non-assigned signals). **Anomeric C atom of the maltose unit. (presented in Klajnert et al., *Chem. Eur. J.* **2008**, *14*, 7030).

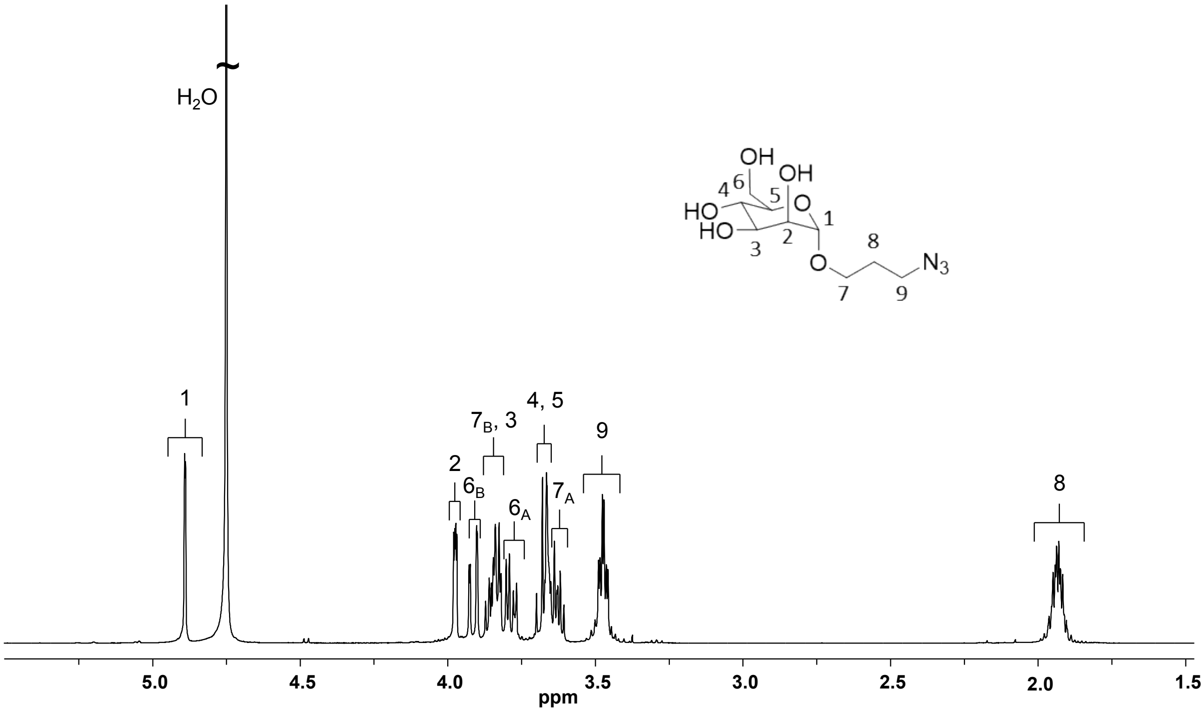

Figure S10. ^{1}H NMR spectrum of 1-azidopropyl-modified mannose in D_2O.

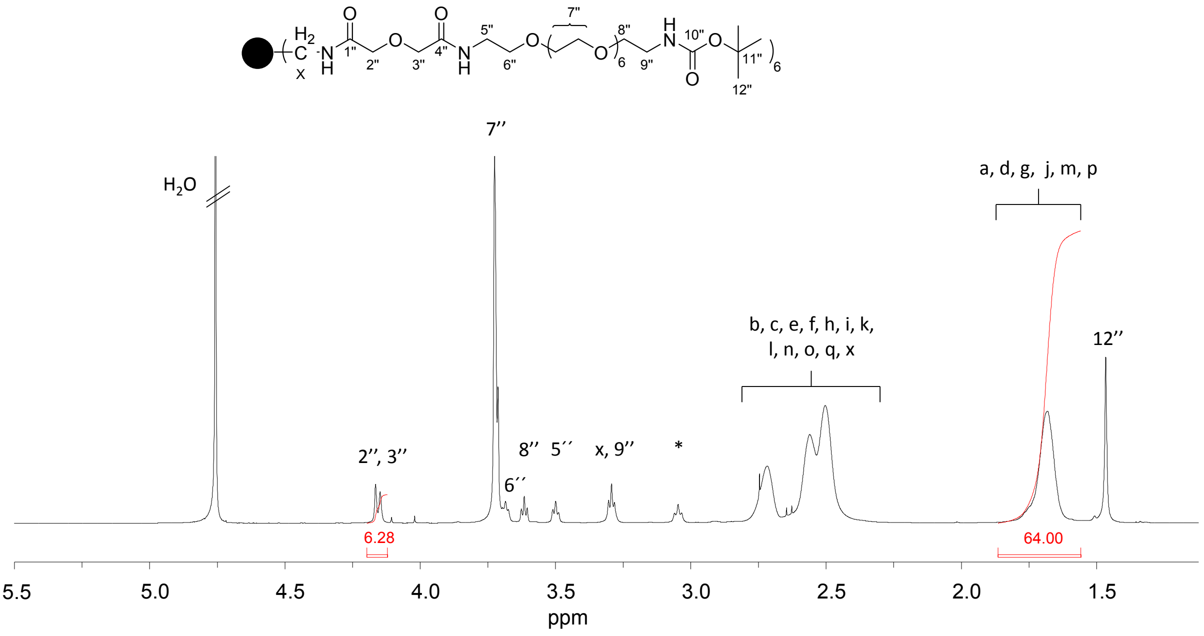

Figure S11. Determination of coupled PEG spacer on PPI scaffold for PPI-PEG (PPI-PEG-II in Table S7). As described under "**Determination of coupled PEG-Spacer on OS-PEG and OS-PEG-Man"** in SI, protons 2´´ and 3´´ represent 1 proton for one coupled PEG spacer, while protons a, d, g, j, m and p represent internal protons of the PPI scaffold which correspond to 64 amino groups. Thus, protons of PPI scaffold were calibrated with 64. With this a direct correlation to the number of coupled PEG spacer via

protons 2´´ and 3´´ is given. Assignment of a-q for PPI-G4 scaffold is adapted from former publication (Klajnert et al., *Chem. Eur. J.* **2008**, *14*, 7030), which is presented in Figure S9.

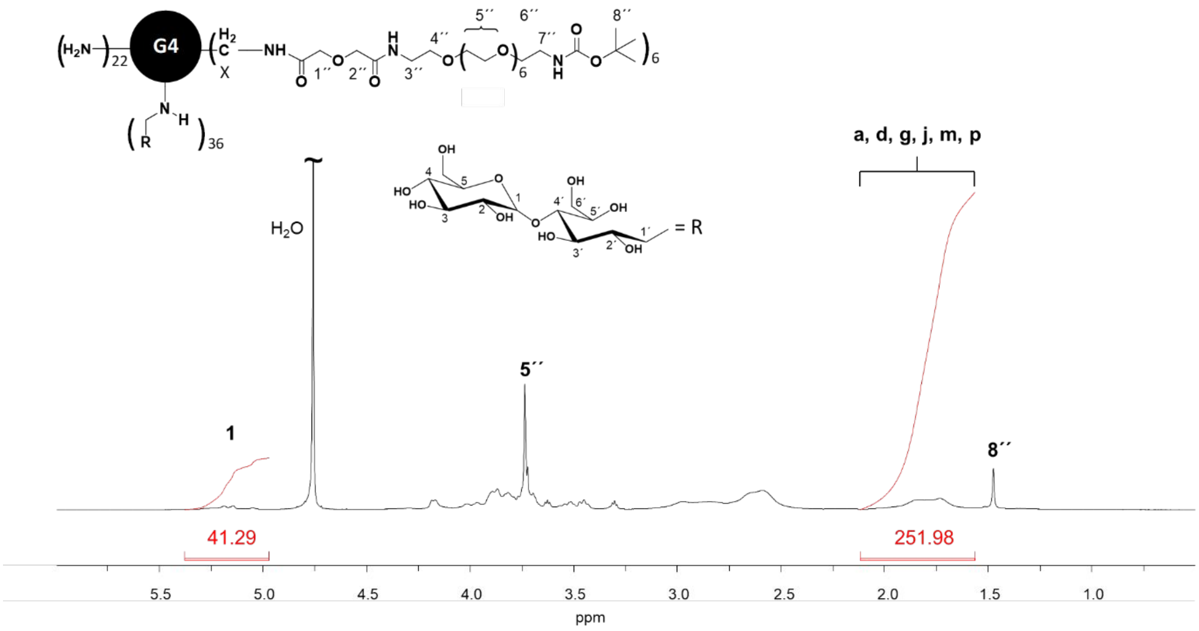

Figure S12. Determination of maltose units attached on PPI scaffold for PPI-Mal-PEG. There is a direct correlation of the anomeric proton 1 of reduced maltose, chemically coupled to the primary amino surface groups of PPI dendrimers, and the internal protons a, d, g, j, m, and p of the PPI scaffold. Protons of PPI scaffold are calibrated with 252 protons. Thus, there is direct read out of coupled maltose units of about 41-42. This approach was described by Klajnert and coworkers (*Chem. Eur. J.* **2008**, *14*, 7030). Further details on the signal assignment for PPI-Mal-PEG is presented in Figure S3, while assignment of PPI scaffold is presented in Figure S9.

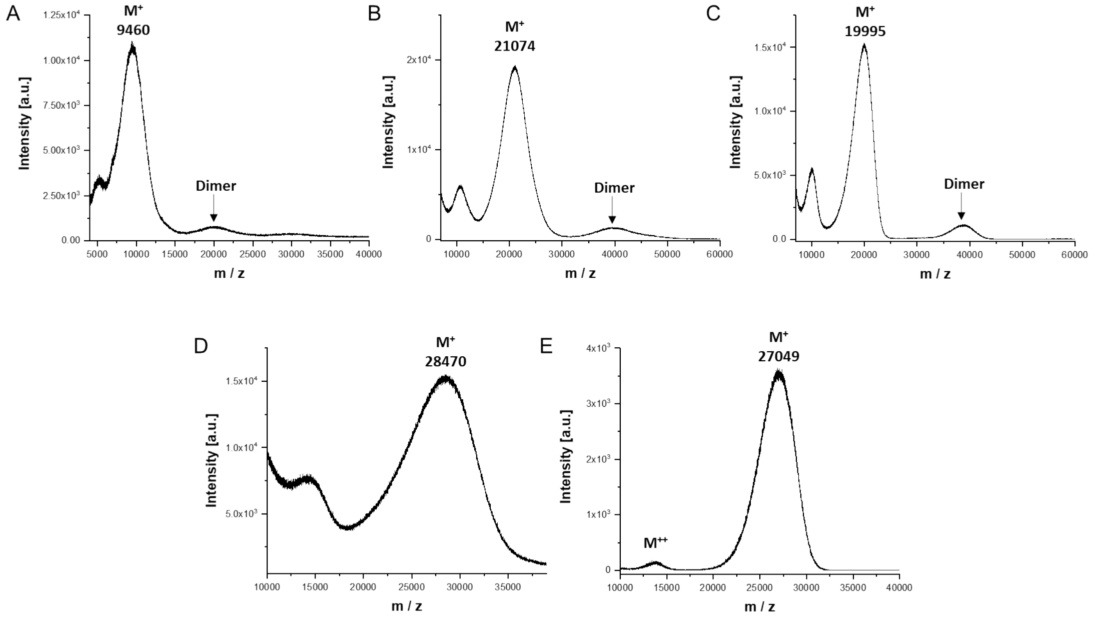

Figure S13. MALDI-TOF spectra of OS-PEG-Man, including their precursors. (A) PPI-PEG-I. (B) PPI-Mal-PEG-I. (C) PPI-Mal-PEG-I-NH_2. (D) PPI-PEG-COD-I. (E) OS-PEG-Man. Corresponding values of M_w are also presented in Table S7. M_w values in Table S7 are round up or down. Data for M^+ represents the top of mass peak. M^+ = singled charged. M^{++} = double charged.

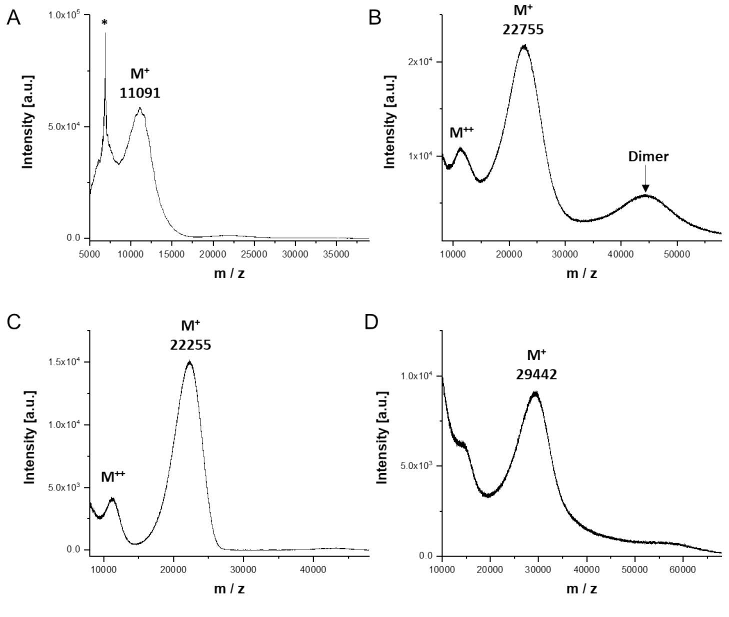

Figure S14. MALDI-TOF spectra of OS-PEG, including their precursors. (A) PPI-PEG-II. (B) PPI-Mal-PEG-II. (C) PPI-Mal-PEG-II-NH_2. (D) OS-PEG. Corresponding values of M_w are also presented in Table S7. M_w values in Table S7 are round up or down. Data for M^+ represents the top of mass peak. * represents degraded PPI-PEG-II.

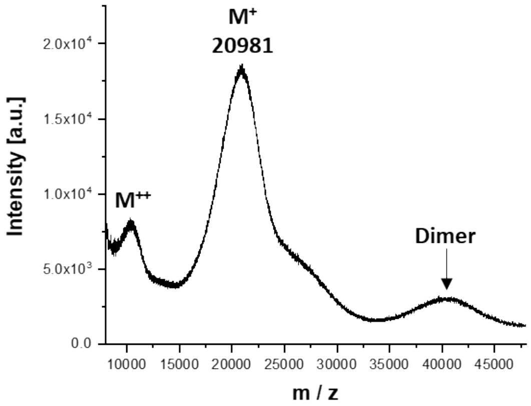

Figure S15. MALDI-TOF spectra of PPI-Mal. Corresponding value of M_w is also presented in Table S7. M_w value in Table S7 is round up. Data for M^+ represents the top of mass peak.

References

[1] Tomalia, D. A.; Rookmaker, M. In *Polymer Data Handbook*, 2nd ed.; Mark, J. E., Ed.; Oxford University Press: Oxford, New York, 2009; pp 979-982.

[2] Firdaus, S.; Geisler, M.; Friedel, P.; Banerjee, S.; Appelhans, D.; Voit, B.; Lederer, A. Glyco-pseudodendrimers on a Polyester Basis: Synthesis and Investigation of Protein-Pseudodendrimer Interaction. *Macromol. Rapid Commun.* **2018**, *39*, 1800364.
